# Computationally Assisted Noncanonical Amino Acid Incorporation

**DOI:** 10.1021/acscentsci.4c01544

**Published:** 2024-12-16

**Authors:** Chengzhu Fang, Wenyuan Xu, Chao Liu, Yulin Chen, Shixian Lin, Wenlong Ding

**Affiliations:** † The Second Affiliated Hospital of Zhejiang University School of Medicine, Life Sciences Institute, Zhejiang University, Hangzhou 310058, China; ‡ Zhejiang Key Laboratory of Molecular Cancer Biology, Center for Life Sciences, Shaoxing Institute, Zhejiang University, Shaoxing 321000, China; § Department of Medical Oncology, State Key Laboratory of Transvascular Implantation Devices, The Second Affiliated Hospital, Zhejiang University School of Medicine, Hangzhou 310058, China; ∥ Institute of Fundamental and Transdisciplinary Research, Zhejiang University, Hangzhou 310058, China; ⊥ Center for Oncology Medicine, the Fourth Affiliated Hospital of School of Medicine, and International School of Medicine, International Institutes of Medicine, Zhejiang University, Yiwu 322000, China

## Abstract

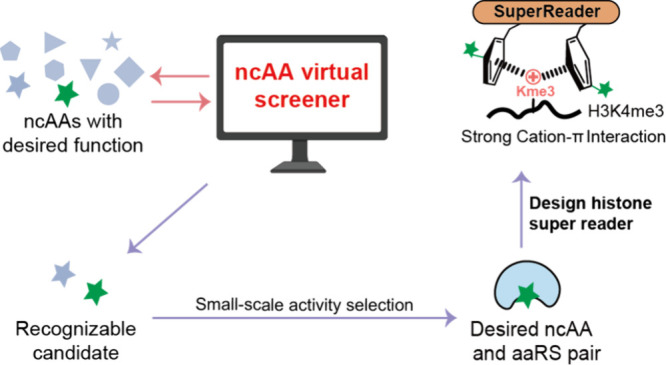

Genetic encoding of noncanonical amino acids (ncAAs) with desired functionalities is an invaluable tool for the study of biological processes and the development of therapeutic drugs. However, existing ncAA incorporation strategies are rather time-consuming and have relatively low success rates. Here, we develop a virtual ncAA screener based on the analysis and modeling of the chemical properties of all reported ncAA substrates to virtually determine the recognition potential of candidate ncAAs. Using this virtual screener, we designed and incorporated several novel Lys and Phe derivatives into proteins for various downstream applications. Among them, the genetic encoding of an electron-rich Phe analog, 3-dimethylamino-phenylalanine, was successfully applied to enhance the cation-π interaction between histone methylation and its reader proteins. Thus, our virtual screener provides a fast and powerful strategy to efficiently incorporate ncAAs with diverse functionalities.

## Abstract 2

We have developed a virtual noncanonical amino acid (ncAA) screener that allows evaluation of the genetic encodability of designed ncAAs with desired functionalities prior to chemical synthesis.

## Introduction

**Figure 1:**
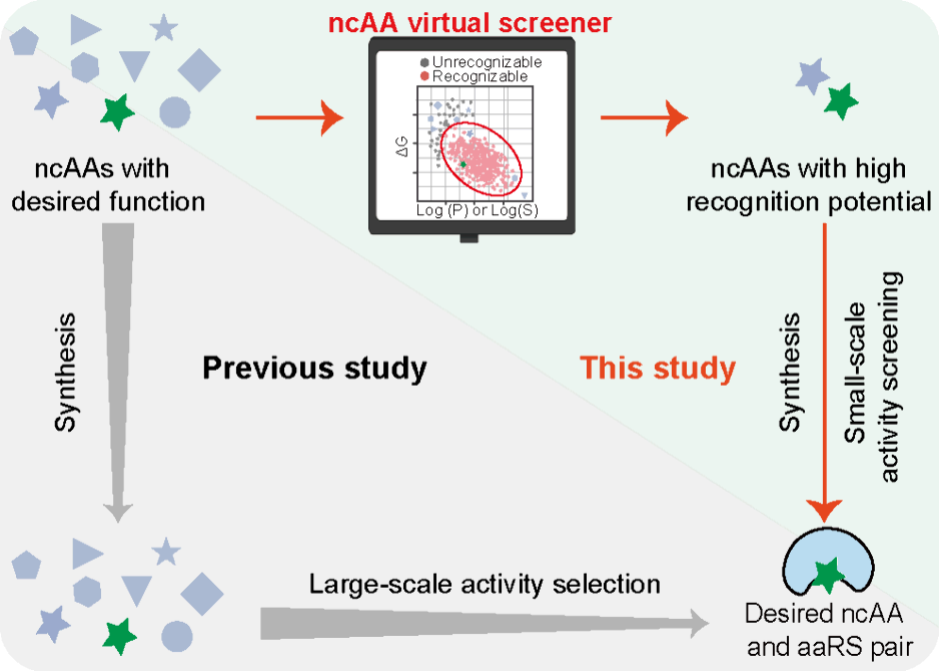
Schematic of computationally assisted noncanonical amino acid incorporation.

Based on the properties of noncanonical amino acids (ncAAs), site-specific incorporation of ncAAs can render proteins with enhanced or novel functions.^1−9^ The genetic code expansion (GCE) strategy utilizes orthogonal aminoacyl-tRNA synthetase (aaRS)/tRNA pairs to recognize ncAAs and site-specifically install them onto the protein of interest by decoding stop codons.^10,11^ Although more than 300 ncAAs^12−14^ have been incorporated and utilized in a variety of fundamental^15−24^ and applied research,^25−30^ the genetic encoding of novel ncAAs with unique physical, chemical, or biological functions remains one of the most interesting and challenging endeavors in the field. For instance, in order to incorporate a desired function into a protein, multiple ncAAs with such functionality had to be designed, chemically synthesized, and screened for active aaRS variants through multiple rounds of positive and negative selections (Figure 1).^31^ These procedures are often very time-consuming and labor-intensive, as it typically takes months or even years for specialized laboratories to incorporate a new functional ncAA. In addition, the success rate of screening for active aaRS variants is also low, resulting in many functional ncAAs not being incorporated (Figure 1). Therefore, there is a pressing need to develop a method that can virtually assess the recognition potential of designed ncAAs prior to initiating chemical synthesis and aaRS selection.

Recently, virtual screening methods have been successfully used to predict whether a given compound is a potential enzyme substrate by analyzing and modeling the properties of all known enzyme substrates.^32,33^ Therefore, we hypothesized that by comprehensively analyzing the properties of all genetically encodable ncAAs in the literature, it would also be useful to develop virtual screening models to predict whether a given ncAA is a potential substrate of aaRS. Here, we systematically analyzed all reported ncAAs of four widely used orthogonal aaRS/tRNA pairs in mammalian cells, including pyrrolysyl-tRNA synthetase/tRNA (PylRS/PylT),^34^ chimeric phenylalanyl-tRNA synthetase/tRNA (chPheRS/chPheT),^35^*E. coli* tyrosyl-tRNA synthetase/tRNA (*Ec*TyrRS/*Ec*TyrT)^36^ and *E. coli* leucyl-tRNA synthetase/tRNA (*Ec*LeuRS/*Ec*LeuT).^37^ By modeling the molecular properties of these ncAAs and their interaction features with aaRS, we developed parameters that are critical for virtually screening the recognition potential of designed ncAA by aaRS (Figure 1). We evaluated our model for the design and incorporation of Lys and Phe derivatives for various downstream applications such as engineering protein post-translational modifications and tuning cation-π interactions.

## Results

We first evaluated the ability of aaRS to recognize a particular ncAA based on its intrinsic chemical properties and its ability to interact with aaRS. The enzyme kinetics of PylRS and TyrRS indicate that the Km value of ncAA is in the mM range.^38−40^ Screening for active aaRS mutants is possible only when the intracellular concentration of the designed ncAA reaches the mM range, which depends on the permeability and solubility of the compound.^4^ Therefore, ncAA with high recognition potential should have relatively high cellular uptake efficiency and solubility to be utilized by aaRS in living cells. In addition, the binding affinity of ncAA for aaRS should be within a certain range; too weak to be bound by aaRS and too strong to efficiently release the aminoacylation product. We envisioned that the chemical properties and binding ability of ncAA can be obtained by analyzing and modeling the properties of all reported ncAAs, both recognizable and unrecognizable for the given aaRSs. These parameters can be packaged as virtual screening filters to evaluate our designed ncAAs and exclude those ncAAs that are unlikely to be incorporated (Figure 1).

**Figure 2:**
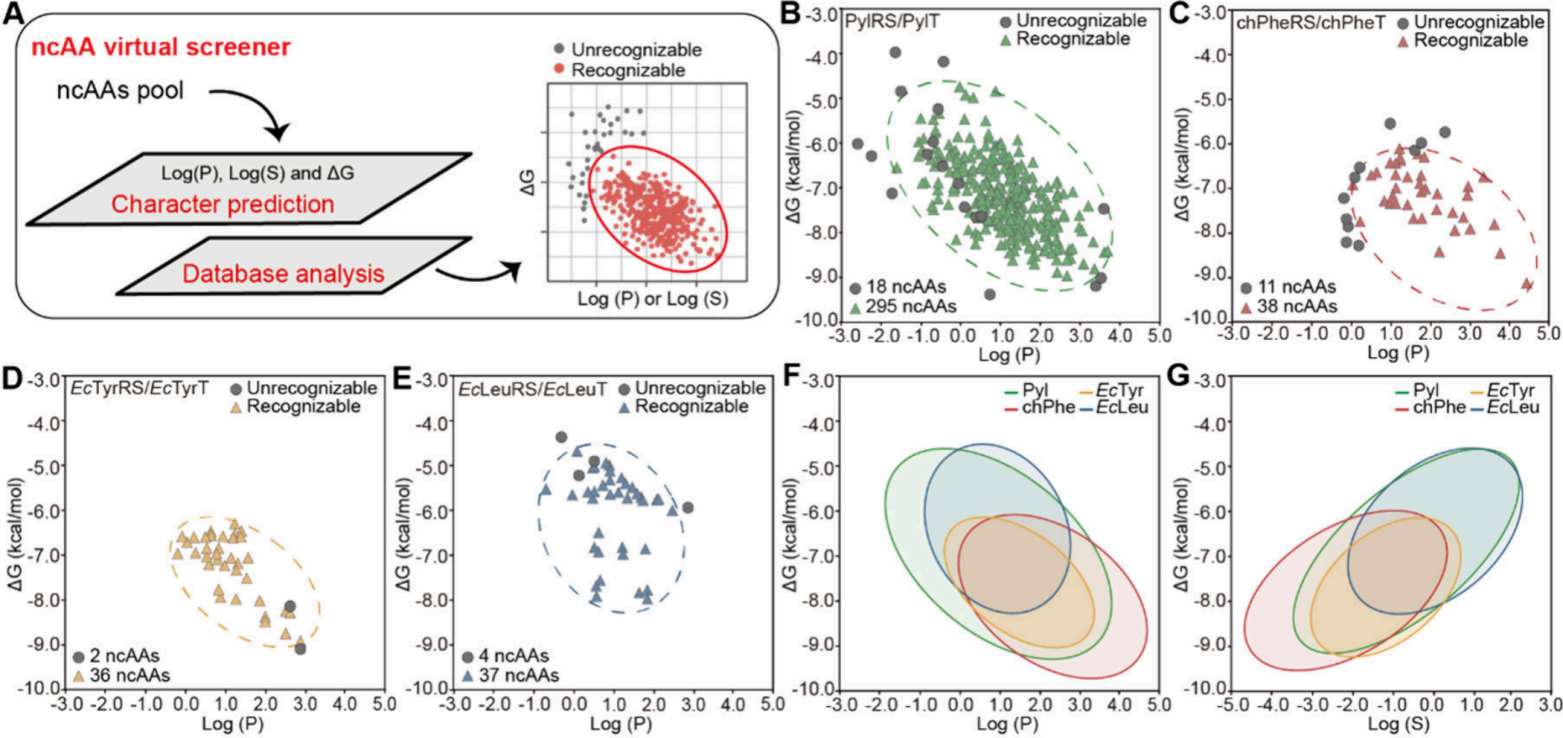
Development of the ncAA virtual screener. (A) Schematic of the construction of virtual ncAA screener. (B)–(E) The virtual ncAA screener based on Log (P) and Δ*G* values for Pyl system (B), chPhe system (C), *Ec*Tyr system (D), *Ec*Leu system (E), respectively. Overlapping of four virtual ncAA screeners based on Log (P) and Δ*G* values (F), and Log (S) and Δ*G* values (G).

To achieve this goal, we initially selected a set of parameters to quantify the recognition of ncAAs by aaRS, including the permeability and the solubility of ncAAs, as well as the affinity of ncAAs for aaRS: (1) The permeability of ncAA was quantified by calculating the n-octanol: water partition coefficient (calculated Log (P)), which was predicted from the chemical structure of the amino acid; (2) the water solubility of ncAA was evaluated by Log (S), which was also predicted from the chemical structure of the ncAA; and (3) the affinity of ncAA by aaRS was assessed by the Gibbs free energy (Δ*G*), which was obtained by molecular docking of ncAA to a given synthetase pocket (Figure S1). To simplify the process, we utilized aaRS mutants with deep substrate binding pockets as the docking receptors. With these established evaluation criteria, we developed a computational program for automated virtual screening, achieving a screening rate of ∼450 compounds per day. Using this automated program, we analyzed these three key parameters for all reported recognizable and unrecognizable ncAAs for their corresponding aaRS types. These parameters can therefore define a spatial boundary that encompasses all recognizable ncAAs and forms a screener for a specific type of aaRS (Figure 2A).

After a systematic survey, the model was built and trained using a comprehensive data set of reported ncAAs, with both recognizable and unrecognizable for several aaRS systems (PylRS (295, 18), chPheRS (38, 11), *Ec*TyrRS (36, 2), and *Ec*LeuRS (37, 4)). Each ncAA was characterized by its Log (P), Log (S), and Δ*G* values, and the data set was considered as a training set for model building. Taking the PylRS as an example, the data were processed by plotting a two-dimensional scatterplot with Δ*G* as the *y*-axis, and Log (P) or Log (S) as the *x*-axis, respectively (Figure 2B, Figure S2A). It is noteworthy that the recognizable ncAAs in the plots are clustered within an elliptical region, while most of the unrecognizable ncAAs are distributed outside of this elliptical region. This phenomenon is particularly evident in the plot of Δ*G* versus Log (P). Therefore, we speculated that if the point of a designed ncAA (defined by its Δ*G* and Log (P) values) lies within this elliptical region, then the PylRS would have a high probability of recognizing this ncAA. The same analysis was performed for the other three aaRS systems, and the results showed a similar distribution pattern (Figure 2B-E and Figure S2). When we merged the plots of all four aaRS systems, they overlapped and had their own unique regions (Figure 2F-G). Most of the ncAAs in the overlapping regions belong to Phe derivatives, which are common substrates for all four systems. The elliptical region of PylRS is the largest, consistent with the fact that PylRS has a wider range of substrates.^12^ The region of chPheRS is located at the bottom right, suggesting that the system can specifically incorporate ncAAs with high hydrophobicity. Indeed, hydrophobic lipidation mimics have recently been successfully incorporated by the chPheRS.^41^ Together, our screener model not only provides the information on whether the designed ncAAs can be recognized, but also suggests their most promising aaRS system.

**Figure 3:**
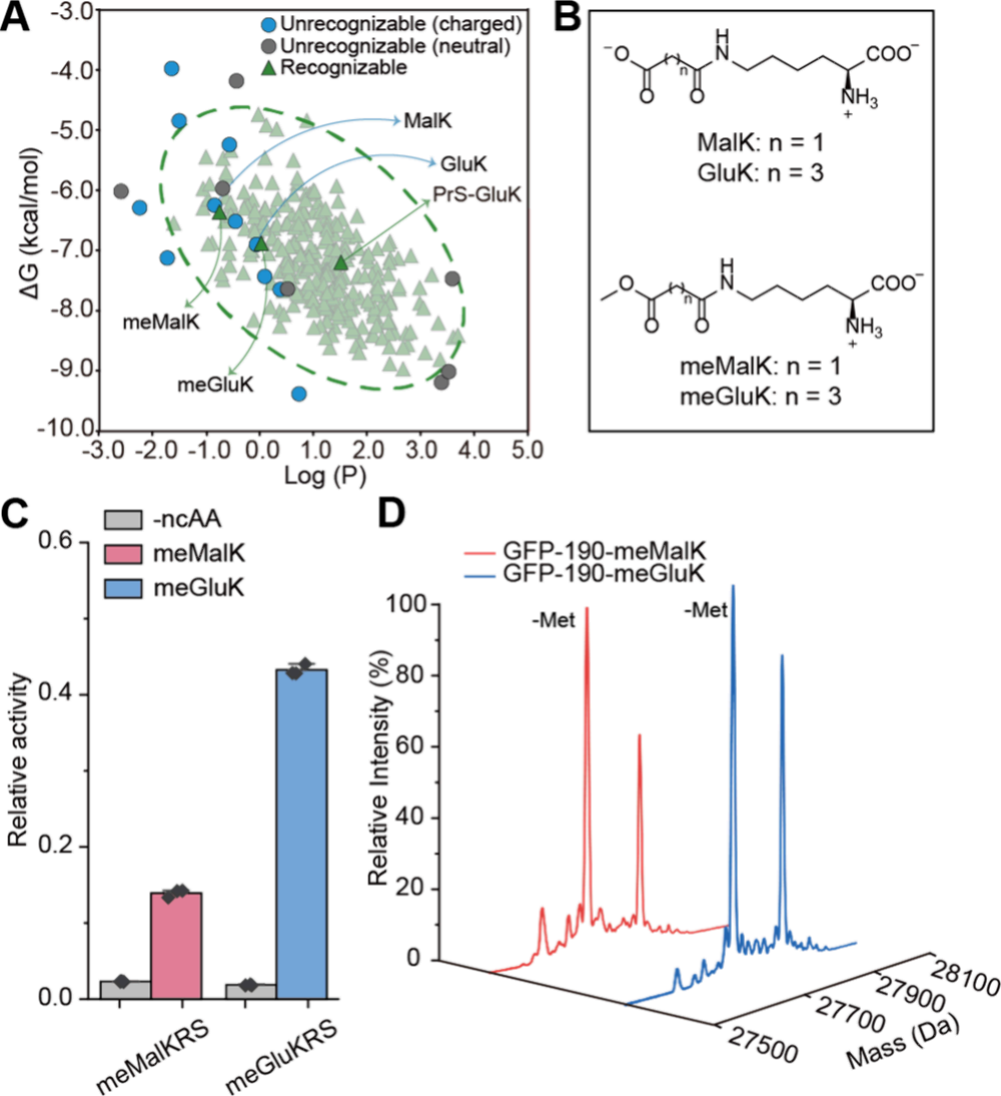
The computer-assisted incorporation of the malonylation lysine and glutamylation lysine. (A) Predicated recognizability of Lys derivatives including MalK, GluK, meMalK, meGluK and PrS-GluK with our established virtual ncAA screener of PylRS. (B) Molecular structure of the Lys derivatives. (C) The amber suppression efficiency was tested by a GFP reporter assay with and without the addition of ncAAs for two variants of PylRS. The fluorescent intensity in each group was normalized by the GFP-WT set as 1. The variants were named as meMalKRS and meGluKRS respectively. The error bars represent ± standard error of the mean from three biologically independent experiments. (D) Mass spectrometry characterization of the fidelity of meMalK and meGluK incorporation into GFP. The expected molecular wight (MW) value of GFP incorporated with meMalK and meGluK was 27836 and 27864 Da, and the observed MW value was 27836 and 27864 Da, respectively. The peaks with N-Met cleavage were also detected.

With the screener in hand, we then leveraged this strategy to incorporate ncAAs with the desired function. We first focused on the malonylated lysine (MalK) and glutamylated lysine (GluK), modifications that invert the charge from +1 to −1. Unfortunately, PylRS is known to prefer amino acids with hydrophobic side chains (Data S1), making it difficult to find active PylRS variants that recognize the negative charge of MalK and GluK. In contrast, we designed methyl ester versions of MalK and GluK, termed meMalK and meGluK, that partially mimic both modifications (Figure 3A-B). Both compounds survived from the PylRS screener and were synthesized in a two-step reaction (Figure S7). Subsequently, a small-scale of MbPylRS mutants were screened using a GFP amber suppression assay in the presence of meMalK and meGluK (Figure S3). The screening yielded two PylRS mutants with high amber suppression efficiencies for meMalK and meGluK, which were dubbed as meMalKRS (C313 V) and meGluKRS (L274A/C313T), respectively (Figure 3C). LC-MS analysis further revealed the high fidelity of meMalK and meGluK incorporation (Figure 3D). Moreover, we found that the PylRS variants also functioned in mammalian cells (Figure S4). During the preparation of our work, a nice work reported the incorporation of GluK via masking the negative charge with thioester (Prs-GluK), which can be removed when incorporated to generate authentic modification in living cells.^42^ And PrS-GluK is also located in the center of the recognition ellipse, demonstrating the feasibility of our computational strategy (Figure 3A).

**Figure 4:**
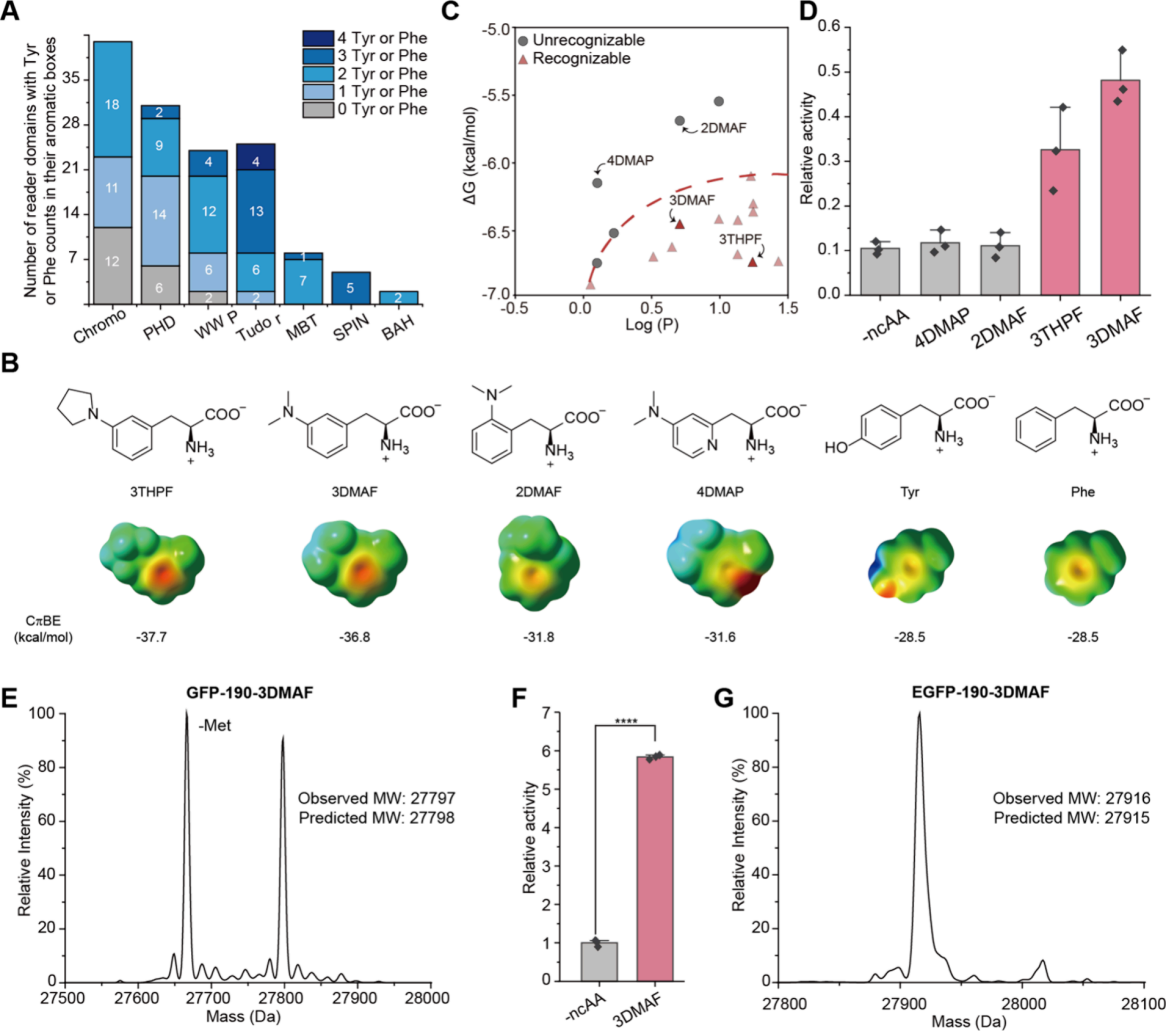
Computer-assisted incorporation of ncAAs with high cation-π interaction potential. (A) Statistics of the presence of Tyr or Phe residues in aromatic boxes of seven types of histone methylation reader domains, including Chromo (chromatin organization modifier), PHD (plant homeodomain), PWWP (domain with a conserved Pro-Trp-Trp-Pro motif), Tudor, MBT (malignant brain tumor), SPIN (Spindlin), and BAH (bromo adjacent homology). (B) Molecular structure of the Tyr and Phe derivatives designed in the study. The electrostatic potential (ESP) maps of the designed derivatives side chain and the cation−π binding energy (CπBE) of the designed derivatives side chain with Na^+^ were calculated and shown. (C) Predicted recognizability of our designed ncAAs with the established virtual ncAA screener of chPheRS. (D) The amber suppression efficiency of different ncAAs was tested by a GFP reporter assay using chPheRS-3 variant. The fluorescent intensity in each group was normalized by the GFP-WT set as 1. Error bars represent ± standard error of the mean of three biologically independent experiments. (E) Mass spectrometry characterization of the fidelity of 3DMAF incorporation into GFP. (F) Flow cytometry analysis of the amber suppression efficiency of 3DMAF incorporation in HEK293T cells. The mean fluorescence intensity ratio of EGFP to mCherry in the absence of 3DMAF was set as 1. Error bars represent the ± standard error of the mean from three biologically independent experiments. Statistical significance was quantified with *t* test (****p **<** 0.0001). (G) Mass spectrometry characterization of the fidelity of 3DMAF incorporation into EGFP in HEK293T.

Cation-π interactions play a crucial role in molecular recognition.^43−45^ And the incorporation of ncAA, which provides strong cation-π interaction, is a promising direction for deciphering the biological function of cation-π interactions.^46,47^ Histone methylation, which occurs at lysine or arginine residues, is an important epigenetic modification that regulates gene expression through various writers, readers and erasers.^48−50^ These proteins contain an aromatic box, typically consisting of two to four aromatic residues (Trp, Tyr, and Phe), which recognizes methylation events through cation-π interactions. Our previous studies have shown that substitution of the key Trp with electron-rich Trp analogs in the aromatic box of the H3Kme3 reader significantly enhances the cation-π interaction, generating Super-Readers with enhanced binding affinity for various applications.^46,47^ However, a similar substitution strategy is lacking for Tyr and Phe residues in the aromatic box, where Tyr and Phe residues are also enriched. Systematic analysis revealed that 85% (116 out of 136) of the aromatic boxes of seven histone methylation reader domains contained at least one Tyr or Phe residue (Figure 4A and Table S1). We therefore hypothesized that replacing these Tyr or Phe with more electron-rich derivatives in the reader domains might also improve the affinity between the histone methylation mark and its reader by enhancing the cation-π interaction.

We then designed several Phe derivatives with strong electron-rich dialkylamino substitution groups. Theoretical calculations indicated that these newly designed ncAAs exhibited higher negative electrostatic potentials and stronger cation-π binding energies than Tyr and Phe (Figure 4B). We then evaluated the recognition potential of these designed ncAAs using the chPheRS screener, as a range of Tyr and Phe derivatives have been efficiently incorporated by this system.^35,41,51^ The analysis showed that 3-dimethylamino-phenylalanine (3DMAF) and 3-(3-tetrahydropyrrol-1-yl-phenyl)alanine (3THPF) had high recognition rates, while 2-dimethylamino-phenylalanine (2DMAF) and 3-(4-dimethylamino-pyridin-2-yl)alanine (4DMAP) did not meet the recognition criteria (Figure 4C). To verify our predicted results, we chemically synthesized these four ncAAs (Figure S8) and screened our previously reported chPheRS mutants for their recognition using the GFP amber suppression assay.^32^ After screening a variety of chPheRS variants, we found that 3DMAF and 3THPF could be recognized by chPheRS-3 (E391D/T467G/A507G), whereas 2DMAF and 4DMAP could not be recognized by these variants (Figure 4D). LC-MS analysis demonstrated that 3DMAF was successfully incorporated with high fidelity, whereas the fidelity of 3THPF incorporation was relatively low (Figure 4E and Figure S5). Since the chimeric Phe translation system is an *E. coli*-mammalian shuttle system, we further tested the amber suppression efficiency of 3DMAF in mammalian cells. HEK293T cells were cotransfected with a plasmid carrying tRNA and the chPheRS-3 as well as a plasmid harboring mCherry-T2A-EGFP 190TAG. Full-length EGFP expression was detected in the presence of 3DMAF by fluorescence-activated cell sorting (FACS) assay (Figure 4F and Figure S6). And LC-MS analysis further showed the high fidelity of 3DMAF incorporation in mammalian cells (Figure 4G).

**Figure 5:**
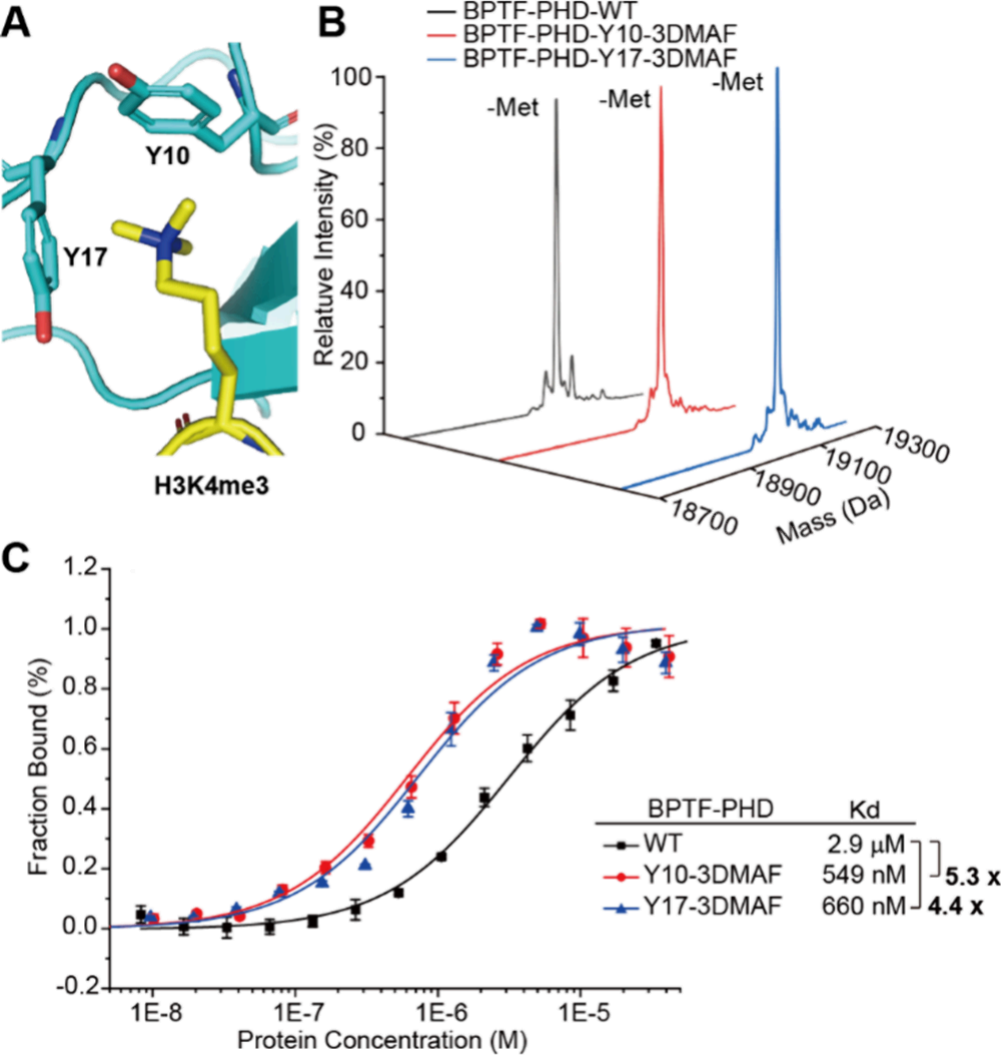
Design of a reader domain with improved histone methylation recognition. (A) Structure of the BPTF-PHD finger (cyan) in complex with the H3K4me3 peptide (yellow) (PDB: 2F6J). The key Tyr residues were showed as sticks. (B) Mass spectrometry characterization of the fidelity of 3DMAF incorporation into BPTF-PHD. The expected molecular mass (MW) value of BPTF-PHD-WT, BPTF-PHD-Y10-3DMAF, and BPTF-PHD-Y17-3DMAF with N-Met cleavage were 19045, 19072, and 19072 Da, and the observed MW value were 19043, 19070, and 19071 Da, respectively. (C) Microscale thermophoretic analysis of BPTF-PHD-WT, BPTF-PHD-Y10-3DMAF and BPTF-PHD-Y17-3DMAF with the H3K4me3 peptide. H3K4me3 was labeled with a fluorescent dye molecule, fluorescein isothiocyanate (FITC). Error bars represent ± standard error of the mean from three biologically independent experiments.

Furthermore, we investigated whether replacing the Tyr residue in the aromatic box with an electron-rich derivative could improve the binding affinity of the reader domain to H3Kme3. We chose the BPTF-PHD domain, a reader of H3K4me3, as an example. Both the wild-type of the BPTF-PHD domain and its variants carrying 3DMAF at either Y10 or Y17 of its aromatic box were expressed with good yield and high fidelity (Figure 5A-B). We then measured the binding affinity of wild-type protein (BPTF-PHD-WT) or protein variants (BPTF-PHD-Y10-3DMAF and BPTF-PHD-Y17-3DMAF) to the H3K4me3 peptide, respectively, using microscale thermophoresis (MST). The binding affinity of BPTF-PHD-WT to H3K4me3 was 2.9 μM, which is consistent with the previously reported results.^52^ Encouragingly, the binding affinities of BPTF-PHD-Y10-3DMAF and BPTF-PHD-Y17-3DMAF to H3K4me3 peptides were 549 nM and 660 nM, respectively, which were 5.3-fold and 4.4-fold higher compared to WT protein (Figure 5C). This result indicated that the genetic encoding of the electron-rich Tyr or Phe derivatives in the aromatic box could also increase the binding affinity to histone methylation and be used together with the electron-rich Trp derivative to design stronger histone methylation Super-Readers.

## Conclusion

In summary, we developed a virtual ncAA screener strategy for virtually evaluating the recognition potential of the designed ncAAs with desired functionality prior to the chemical synthesis of these ncAAs and selection of aaRS. By systematically analyzing the chemical properties of all recognizable and unrecognizable ncAAs reported in the literature, as well as their binding affinities with four aaRS systems, we generated four virtual screeners. We demonstrated that the ncAAs surviving from the virtual screener can be readily incorporated into protein by the small-scale aaRS variant screening. Using this strategy, several Lys and Phe derivatives were designed and successfully incorporated into protein in a site-specific manner, including 3DMAF with high cation-π binding energy. We subsequently demonstrated that substitution of Tyr with 3DMAF in the aromatic box significantly increased the affinity between histone methylation and its reader domains, suggesting that substitution of residues with electron-rich analogs in the binding pocket may be a general strategy for designing high-affinity binders. We anticipate that our prediction models will become more accurate as the library of ncAAs continues to expand, which further accelerates the ncAA recognition process. The generated data will ultimately contribute to the computational design of aaRS to recognize new ncAAs through generative artificial intelligence. Thus, our study provides a cost-effective, time-saving, and efficient strategy to assist in the genetic encoding of functional ncAAs.

## Supporting Information Available

Experimental materials, methods, and supporting figures, including NMR spectra for all compounds (PDF)
The predicted property data for all ncAAs (XLSX)
Transparent Peer Review report available (PDF)

## References

[ref1] Wang Y.; Zhang J.; Han B.; Tan L.; Cai W.; Li Y.; Su Y.; Yu Y.; Wang X.; Duan X.; Wang H.; Shi X.; Wang J.; Yang X.; Liu T. Noncanonical amino acids as doubly bio-orthogonal handles for one-pot preparation of protein multiconjugates. Nat. Commun. 2023, 14, 974 10.1038/s41467-023-36658-y. 36810592 PMC9944564

[ref2] Longwitz L.; Leveson-Gower R. B.; Rozeboom H. J.; Thunnissen A. W. H.; Roelfes G. Boron catalysis in a designer enzyme. Nature 2024, 629, 824–829. 10.1038/s41586-024-07391-3. 38720081

[ref3] Chatterjee A.; Guo J.; Lee H. S.; Schultz P. G. A genetically encoded fluorescent probe in mammalian cells. J. Am. Chem. Soc. 2013, 135, 12540–3. 10.1021/ja4059553. 23924161 PMC3783214

[ref4] Huguenin-Dezot N.; Alonzo D. A.; Heberlig G. W.; Mahesh M.; Nguyen D. P.; Dornan M. H.; Boddy C. N.; Schmeing T. M.; Chin J. W. Trapping biosynthetic acyl-enzyme intermediates with encoded 2,3-diaminopropionic acid. Nature 2019, 565, 112–117. 10.1038/s41586-018-0781-z. 30542153 PMC6436733

[ref5] Zhang J.; Wang X.; Huang Q.; Ye J.; Wang J. Genetically Encoded Epoxide Warhead for Precise and Versatile Covalent Targeting of Proteins. J. Am. Chem. Soc. 2024, 146, 16173–16183. 10.1021/jacs.4c03974. 38819260 PMC11177858

[ref6] Wang N.; Yang B.; Fu C.; Zhu H.; Zheng F.; Kobayashi T.; Liu J.; Li S.; Ma C.; Wang P. G.; Wang Q.; Wang L. Genetically Encoding Fluorosulfate-l-tyrosine To React with Lysine, Histidine, and Tyrosine via SuFEx in Proteins in Vivo. J. Am. Chem. Soc. 2018, 140, 4995–4999. 10.1021/jacs.8b01087. 29601199 PMC6031228

[ref7] Qianzhu H.; Abdelkader E. H.; Otting G.; Huber T. Genetic Encoding of Fluoro-l-tryptophans for Site-Specific Detection of Conformational Heterogeneity in Proteins by NMR Spectroscopy. J. Am. Chem. Soc. 2024, 146, 13641–13650. 10.1021/jacs.4c03743. 38687675

[ref8] Jang H. S.; Jana S.; Blizzard R. J.; Meeuwsen J. C.; Mehl R. A. Access to Faster Eukaryotic Cell Labeling with Encoded Tetrazine Amino Acids. J. Am. Chem. Soc. 2020, 142, 7245–7249. 10.1021/jacs.9b11520. 32251579 PMC7771912

[ref9] Ding W.; Yu W.; Chen Y.; Lao L.; Fang Y.; Fang C.; Zhao H.; Yang B.; Lin S. Rare codon recoding for efficient noncanonical amino acid incorporation in mammalian cells. Science 2024, 384, 1134–1142. 10.1126/science.adm8143. 38843324

[ref10] Wang L.; Brock A.; Herberich B.; Schultz P. G. Expanding the Genetic Code of Escherichia coli. Science 2001, 292, 498–500. 10.1126/science.1060077. 11313494

[ref11] de la Torre D.; Chin J. W. Reprogramming the genetic code. Nat. Rev. Genet. 2021, 22, 169–184. 10.1038/s41576-020-00307-7. 33318706

[ref12] Koch N. G.; Budisa N. Evolution of Pyrrolysyl-tRNA Synthetase: From Methanogenesis to Genetic Code Expansion. Chem. Rev. 2024, 124, 9580–9608. 10.1021/acs.chemrev.4c00031. 38953775 PMC11363022

[ref13] Jann C.; Giofre S.; Bhattacharjee R.; Lemke E. A. Cracking the Code: Reprogramming the Genetic Script in Prokaryotes and Eukaryotes to Harness the Power of Noncanonical Amino Acids. Chem. Rev. 2024, 124, 10281–10362. 10.1021/acs.chemrev.3c00878. 39120726 PMC11441406

[ref14] Icking L. S.; Riedlberger A. M.; Krause F.; Widder J.; Frederiksen A. S.; Stockert F.; Spadt M.; Edel N.; Armbruster D.; Forlani G.; Franchini S.; Kaas P.; Kirpat Konak B. M.; Krier F.; Lefebvre M.; Mazraeh D.; Ranniger J.; Gerstenecker J.; Gescher P.; Voigt K.; Salavei P.; Gensch N.; Di Ventura B.; Ozturk M. A. iNClusive: a database collecting useful information on non-canonical amino acids and their incorporation into proteins for easier genetic code expansion implementation. Nucleic Acids Res. 2024, 52, D476–D482. 10.1093/nar/gkad1090. 37986218 PMC10767842

[ref15] Weng Y.; Chen W.; Kong Q.; Wang R.; Zeng R.; He A.; Liu Y.; Mao Y.; Qin Y.; Ngai W. S. C.; Zhang H.; Ke M.; Wang J.; Tian R.; Chen P. R. DeKinomics pulse-chases kinase functions in living cells. Nat. Chem. Biol. 2024, 20, 615–623. 10.1038/s41589-023-01497-x. 38167916

[ref16] Zang J.; Chen Y.; Liu C.; Hu L.; Zhao H.; Ding W.; Yuan Y.; Lin S. Genetic code expansion reveals aminoacylated lysine ubiquitination mediated by UBE2W. Nat. Struct. Mol. Biol. 2023, 30, 62–71. 10.1038/s41594-022-00866-9. 36593310

[ref17] Fottner M.; Brunner A. D.; Bittl V.; Horn-Ghetko D.; Jussupow A.; Kaila V. R. I.; Bremm A.; Lang K. Site-specific ubiquitylation and SUMOylation using genetic-code expansion and sortase. Nat. Chem. Biol. 2019, 15, 276–284. 10.1038/s41589-019-0227-4. 30770915

[ref18] Peng T.; Das T.; Ding K.; Hang H. C. Functional analysis of protein post-translational modifications using genetic codon expansion. Protein Sci. 2023, 32, e4618 10.1002/pro.4618. 36883310 PMC10031814

[ref19] Gan Q.; Fan C. Orthogonal Translation for Site-Specific Installation of Post-translational Modifications. Chem. Rev. 2024, 124, 2805–2838. 10.1021/acs.chemrev.3c00850. 38373737 PMC11230630

[ref20] Ding W.; Zhao H.; Chen Y.; Lin S. New Strategies for Probing the Biological Functions of Protein Post-translational Modifications in Mammalian Cells with Genetic Code Expansion. Acc. Chem. Res. 2023, 56, 2827–2837. 10.1021/acs.accounts.3c00460. 37793174

[ref21] Yu M.; Heidari M.; Mikhaleva S.; Tan P. S.; Mingu S.; Ruan H.; Reinkemeier C. D.; Obarska-Kosinska A.; Siggel M.; Beck M.; Hummer G.; Lemke E. A. Visualizing the disordered nuclear transport machinery in situ. Nature 2023, 617, 162–169. 10.1038/s41586-023-05990-0. 37100914 PMC10156602

[ref22] Qin F.; Li B.; Wang H.; Ma S.; Li J.; Liu S.; Kong L.; Zheng H.; Zhu R.; Han Y.; Yang M.; Li K.; Ji X.; Chen P. R. Linking chromatin acylation mark-defined proteome and genome in living cells. Cell 2023, 186, 1066–1085. 10.1016/j.cell.2023.02.007. 36868209

[ref23] Li Y.; Su Y.; Wang H.; Xie Y.; Wang X.; Chang L.; Jing Y.; Zhang J.; Ma J. A.; Jin H.; Lou X.; Peng Q.; Liu T. Computation-Guided Discovery of Diazole Monosubstituted Tetrazines as Optimal Bioorthogonal Tools. J. Am. Chem. Soc. 2024, 146, 26884–26896. 10.1021/jacs.4c07958. 39164893

[ref24] Ding W.; Gu J.; Xu W.; Wu J.; Huang Y.; Zhang S.; Lin S. The Biosynthesis and Applications of Protein Lipidation. Chem. Rev. 2024, 124, 12176 10.1021/acs.chemrev.4c00419. 39441663

[ref25] Pigula M.; Lai Y. C.; Koh M.; Diercks C. S.; Rogers T. F.; Dik D. A.; Schultz P. G. An unnatural amino acid dependent, conditional Pseudomonas vaccine prevents bacterial infection. Nat. Commun. 2024, 15, 6766 10.1038/s41467-024-50843-7. 39117651 PMC11310302

[ref26] Axup J. Y.; Bajjuri K. M.; Ritland M.; Hutchins B. M.; Kim C. H.; Kazane S. A.; Halder R.; Forsyth J. S.; Santidrian A. F.; Stafin K.; Lu Y.; Tran H.; Seller A. J.; Biroc S. L.; Szydlik A.; Pinkstaff J. K.; Tian F.; Sinha S. C.; Felding-Habermann B.; Smider V. V.; Schultz P. G. Synthesis of site-specific antibody-drug conjugates using unnatural amino acids. Proc. Natl. Acad. Sci. U. S. A. 2012, 109, 16101–6. 10.1073/pnas.1211023109. 22988081 PMC3479532

[ref27] Ptacin J. L.; Caffaro C. E.; Ma L.; San Jose Gall K. M.; Aerni H. R.; Acuff N. V.; Herman R. W.; Pavlova Y.; Pena M. J.; Chen D. B.; Koriazova L. K.; Shawver L. K.; Joseph I. B.; Milla M. E. An engineered IL-2 reprogrammed for anti-tumor therapy using a semi-synthetic organism. Nat. Commun. 2021, 12, 4785 10.1038/s41467-021-24987-9. 34373459 PMC8352909

[ref28] Sigal M.; Matsumoto S.; Beattie A.; Katoh T.; Suga H. Engineering tRNAs for the Ribosomal Translation of Non-proteinogenic Monomers. Chem. Rev. 2024, 124, 6444–6500. 10.1021/acs.chemrev.3c00894. 38688034 PMC11122139

[ref29] Li Q.; Chen Q.; Klauser P. C.; Li M.; Zheng F.; Wang N.; Li X.; Zhang Q.; Fu X.; Wang Q.; Xu Y.; Wang L. Developing Covalent Protein Drugs via Proximity-Enabled Reactive Therapeutics. Cell 2020, 182, 85–97. 10.1016/j.cell.2020.05.028. 32579975

[ref30] Alcala-Torano R.; Islam M.; Cika J.; Lam K. H.; Jin R.; Ichtchenko K.; Shoemaker C. B.; Van Deventer J. A. Yeast Display Enables Identification of Covalent Single-Domain Antibodies against Botulinum Neurotoxin Light Chain A. ACS Chem. Biol. 2022, 17, 3435–3449. 10.1021/acschembio.2c00574. 36459441 PMC10065152

[ref31] Wang Q.; Parrish A. R.; Wang L. Expanding the genetic code for biological studies. Chem. Biol. 2009, 16, 323–36. 10.1016/j.chembiol.2009.03.001. 19318213 PMC2696486

[ref32] Kroll A.; Ranjan S.; Engqvist M. K. M.; Lercher M. J. A general model to predict small molecule substrates of enzymes based on machine and deep learning. Nat. Commun. 2023, 14, 2787 10.1038/s41467-023-38347-2. 37188731 PMC10185530

[ref33] Pertusi D. A.; Moura M. E.; Jeffryes J. G.; Prabhu S.; Walters Biggs B.; Tyo K. E. J. Predicting novel substrates for enzymes with minimal experimental effort with active learning. Metab Eng. 2017, 44, 171–181. 10.1016/j.ymben.2017.09.016. 29030274 PMC7055960

[ref34] Wan W.; Tharp J. M.; Liu W. R. Pyrrolysyl-tRNA synthetase: an ordinary enzyme but an outstanding genetic code expansion tool. Biochim. Biophys. Acta 2014, 1844, 1059–70. 10.1016/j.bbapap.2014.03.002. 24631543 PMC4016821

[ref35] Ding W.; Zhao H.; Chen Y.; Zhang B.; Yang Y.; Zang J.; Wu J.; Lin S. Chimeric design of pyrrolysyl-tRNA synthetase/tRNA pairs and canonical synthetase/tRNA pairs for genetic code expansion. Nat. Commun. 2020, 11, 3154 10.1038/s41467-020-16898-y. 32572025 PMC7308279

[ref36] Sakamoto K.; Hayashi A.; Sakamoto A.; Kiga D.; Nakayama H.; Soma A.; Kobayashi T.; Kitabatake M.; Takio K.; Saito K.; Shirouzu M.; Hirao I.; Yokoyama S. Site-specific incorporation of an unnatural amino acid into proteins in mammalian cells. Nucleic Acids Res. 2002, 30, 4692–9. 10.1093/nar/gkf589. 12409460 PMC135798

[ref37] Wu N.; Deiters A.; Cropp T. A.; King D.; Schultz P. G. A genetically encoded photocaged amino acid. J. Am. Chem. Soc. 2004, 126, 14306–7. 10.1021/ja040175z. 15521721

[ref38] Suzuki T.; Miller C.; Guo L. T.; Ho J. M. L.; Bryson D. I.; Wang Y. S.; Liu D. R.; Soll D. Crystal structures reveal an elusive functional domain of pyrrolysyl-tRNA synthetase. Nat. Chem. Biol. 2017, 13, 1261–1266. 10.1038/nchembio.2497. 29035363 PMC5698177

[ref39] Rauch B. J.; Porter J. J.; Mehl R. A.; Perona J. J. Improved Incorporation of Noncanonical Amino Acids by an Engineered tRNA(Tyr) Suppressor. Biochemistry 2016, 55, 618–28. 10.1021/acs.biochem.5b01185. 26694948 PMC4959848

[ref40] Guo L. T.; Wang Y. S.; Nakamura A.; Eiler D.; Kavran J. M.; Wong M.; Kiessling L. L.; Steitz T. A.; O’Donoghue P.; Soll D. Polyspecific pyrrolysyl-tRNA synthetases from directed evolution. Proc. Natl. Acad. Sci. U. S. A. 2014, 111, 16724–9. 10.1073/pnas.1419737111. 25385624 PMC4250173

[ref41] Ding W.; Liu C.; Chen Y.; Gu J.; Fang C.; Hu L.; Zhang L.; Yuan Y.; Feng X. H.; Lin S. Computational design and genetic incorporation of lipidation mimics in living cells. Nat. Chem. Biol. 2024, 20, 42–51. 10.1038/s41589-023-01400-8. 37563455

[ref42] Weyh M.; Jokisch M. L.; Nguyen T. A.; Fottner M.; Lang K. Deciphering functional roles of protein succinylation and glutarylation using genetic code expansion. Nat. Chem. 2024, 16, 913–921. 10.1038/s41557-024-01500-5. 38531969 PMC11164685

[ref43] Salonen L. M.; Ellermann M.; Diederich F. Aromatic rings in chemical and biological recognition: energetics and structures. Angew.Chem., Int. Ed. 2011, 50, 4808–42. 10.1002/anie.201007560. 21538733

[ref44] Dougherty D. A. The Cation-π Interaction. Acc. Chem. Res. 2013, 46, 885–893. 10.1021/ar300265y. 23214924 PMC3957424

[ref45] Mahadevi A. S.; Sastry G. N. Cation-pi interaction: its role and relevance in chemistry, biology, and material science. Chem. Rev. 2013, 113, 2100–38. 10.1021/cr300222d. 23145968

[ref46] Zhao H.; Liu C.; Ding W.; Tang L.; Fang Y.; Chen Y.; Hu L.; Yuan Y.; Fang D.; Lin S. Manipulating Cation-pi Interactions with Genetically Encoded Tryptophan Derivatives. J. Am. Chem. Soc. 2022, 144, 6742–6748. 10.1021/jacs.1c12944. 35380832

[ref47] Zhao H.; Tang L.; Fang Y.; Liu C.; Ding W.; Zang S.; Chen Y.; Xu W.; Yuan Y.; Fang D.; Lin S. Manipulating Cation-pi Interactions of Reader Proteins in Living Cells with Genetic Code Expansion. J. Am. Chem. Soc. 2023, 145, 16406–16416. 10.1021/jacs.3c02293. 37432680

[ref48] Ruthenburg A. J.; Allis C. D.; Wysocka J. Methylation of Lysine 4 on Histone H3: Intricacy of Writing and Reading a Single Epigenetic Mark. Mol. Cell 2007, 25, 15–30. 10.1016/j.molcel.2006.12.014. 17218268

[ref49] Cornett E. M.; Ferry L.; Defossez P.-A.; Rothbart S. B. Lysine Methylation Regulators Moonlighting outside the Epigenome. Mol. Cell 2019, 75, 1092–1101. 10.1016/j.molcel.2019.08.026. 31539507 PMC6756181

[ref50] Greer E. L.; Shi Y. Histone methylation: a dynamic mark in health, disease and inheritance. Nat. Rev. Genet. 2012, 13, 343–357. 10.1038/nrg3173. 22473383 PMC4073795

[ref51] Zhao H.; Ding W.; Zang J.; Yang Y.; Liu C.; Hu L.; Chen Y.; Liu G.; Fang Y.; Yuan Y.; Lin S. Directed-evolution of translation system for efficient unnatural amino acids incorporation and generalizable synthetic auxotroph construction. Nat. Commun. 2021, 12, 7039 10.1038/s41467-021-27399-x. 34857769 PMC8639764

[ref52] Li H.; Ilin S.; Wang W.; Duncan E. M.; Wysocka J.; Allis C. D.; Patel D. J. Molecular basis for site-specific read-out of histone H3K4me3 by the BPTF PHD finger of NURF. Nature 2006, 442, 91–95. 10.1038/nature04802. 16728978 PMC4690523

