# Supplementary material for: Computationally Assisted Noncanonical Amino Acid Incorporation
Source: ACS Cent Sci. 2024 Dec 16;11(1):84–90. doi: 10.1021/acscentsci.4c01544 (PMC11758377; doi:10.1021/acscentsci.4c01544)
Supplement: Supplementary file 1 — oc4c01544_si_001.pdf [file oc4c01544_si_001.pdf]

Supplementary Information for:

Computationally assisted noncanonical amino acid incorporation

Chengzhu Fang[1,2†], Wenyuan Xu[1,2†], Chao Liu[1,2†], Yulin Chen[1,2], Shixian Lin[1,2,3,4,*], and Wenlong Ding[1,2,5,*]

*[1]The Second Affiliated Hospital of Zhejiang University School of Medicine, Life Sciences Institute, Zhejiang University, Hangzhou 310058, China. [2]Zhejiang Provincial Key Laboratory for Cancer Molecular Cell Biology, Center for Life Sciences, Shaoxing Institute, Zhejiang University, Shaoxing 321000, China. [3]Department of Medical Oncology, State Key Laboratory of Transvascular Implantation Devices, The Second Affiliated Hospital, Zhejiang University School of Medicine, Hangzhou 310058, China. [4]Institute of Fundamental and Transdisciplinary Research, Zhejiang University, Hangzhou 310058, China. [5]Center for Oncology Medicine, the Fourth Affiliated Hospital of School of Medicine, and International School of Medicine, International Institutes of Medicine, Zhejiang University, Yiwu, China, 322000. *E-mail: sxlin@zju.edu.cn, longwending@163.com*

➢ General Methods

➢ Supplementary Table and Figure

Supplementary Table 1. The aromatic boxes of lysine methylation reader domains.

Supplementary Figure 1. The workflow for the calculation of chemical properties of ncAA.

Supplementary Figure 2. The virtual ncAA screeners based on Log (S) and ΔG values.

Supplementary Figure 3. The amber suppression efficiency of aaRS mutants for ncAAs.

Supplementary Figure 4. The amber suppression efficiency of meGluK system in mammalian cells.

Supplementary Figure 5. The mass spectrometry characterization of the fidelity of 3THPF incorporation into GFP by chPheRS-3.

Supplementary Figure 6. The amber suppression efficiency of 3DMAF system in mammalian cells.

Supplementary Figure 7-8. Chemical synthesis of ncAAs.

Supplementary Figure 9-24. NMR data of the synthetic compounds.

➢ References

- **General Methods**

Reagents

Unless otherwise stated, all commercial reagents were used directly without further purification. Primers and genes were synthesized by Tsingke Biotech. The $H3_{1\text{-}14}$K4me3-FITC peptides were synthesized by KS-V Peptide Biological Technology Co., Ltd (Hefei, China) by total chemical synthesis through standard solid phase peptide synthesis (with >95% purity).

Instrumentations

OD_{600} and fluorescence intensity were acquired with Bio Tek Synergy NEO2. Thermophoretic analysis of protein binding affinity was acquired with Monolith NT.115 and data were processed with NT Analysis Software. FACS data were collected by Beckman CytoFlex S and processed with FlowJo. LC-MS analysis was performed on a SCIEX Triple TOF 6600 MS System. All NMR spectra were measured on Varian Mercury-500 spectrometers.

Strains

DH5α and DH10B strains were used in this study.

Cell culture procedure

HEK 293T cells were maintained in an exponential growth as a monolayer in Dulbecco’s Modified Eagle Medium (DMEM, Thermo Fisher Scientific), high-glucose, 10% fetal bovine serum (FBS, Thermo Fisher Scientific), 1% penicillin-streptomycin solution (Gibco), and incubated at 37°C in 5% CO_2.

The ncAA database preparation

For the Pyl, *Ec*Leu, and *Ec*Tyr systems, all ncAAs (recognizable and unrecognizable) were obtained from literatures published before 2024. Given that unrecognizable ncAAs are less commonly reported, some unrecognizable ncAAs tested in our lab were also included in the evaluation dataset. We used ChemDraw Professional 15.0 to draw and standardize the chemical formula format of all ncAAs. Subsequently, we imported all ncAAs into ChemDraw 3D, minimized the energy using MM2 force field method and saved them as SDF (structure data file) format.

The prediction of Log (P) and Log (S)

The Log (S) of the ncAAs was predicted using Molecule Network in Chem3D. The Log (P) of ncAAs was calculated by the python package RDKit using their SMILES (simplified molecular input line entry specification) strings. The SMILES format of all ncAAs was transformed from the SDF files using the python package RDKit. Molecules containing ferrocene and boron atoms were filtered out to ensure that the computational pipeline was clear. All the above prediction calculations were performed in one batch.

The prediction of aaRS binding affinity for ncAA

The ncAAs were docked into aaRS mutants with deep substrate binding pocket using AutoDock Vina 1.1.2 software with default parameters.[1] The structures of chPheRS mutant (T467G/M490G/A507G), *Ec*LeuRS mutant (M40G/Y499G/Y527A/H537G) and *Ec*TyrRS mutant (Y37G/L71V/D182G/F183M/L186A) were simulated with SWISS-MODEL. These simulated structures and PylRS mutant structure (Y306A/Y384F, accession no. 6AAC) were processed with AutoDockTools 1.5.6 software and set as receptors in the docking.[2] The docking boxes and centers of each standard pocket were listed as follows: PylRS pocket centered at [46.408, 136.665, -10.024] with a docking box of 20 × 20 × 20 Å^3; *Ec*LeuRS pocket centered at [3.988, 36.283, 75.154] with a docking box of 20 × 20 × 20 Å^3; *Ec*TyrRS pocket centered at [10.041, 29.472, 16.353] with a docking box of 20 × 20 × 20 Å^3; chPheRS pocket centered at [5.121, 12.148, 14.981] with a docking box of 20 × 20 × 20 Å^3. The average ΔG of the docking was chosen to evaluate the binding affinity of ncAA to aaRS.

Chemical synthesis

***tert*-butyl N^2-(*tert*-butoxycarbonyl)-N^6-(3-methoxy-3-oxopropanoyl)-*L*-lysinate (3a).** Compound **2a** (628 μL, 6 mmol), hydroxybenzotriazole (HOBt) (973 mg, 7.2 mmol), 1-ethyl-3-(3-dimethylaminopropyl)carbodiimide (EDCI) (1.4 g, 7.2 mmol) and *N*,*N*-Diisopropylethylamine (DIPEA) (4.37 mL, 25 mmol) were dissolved in dry tetrahydrofuran (THF). After 20 minutes, a solution of compound **1** (1.5 g, 5 mmol) in dry THF was added. The mixture was allowed to stir overnight. After the starting material was consumed completely which was detected by TLC, water

was added to quench the reaction and extracted with ethyl acetate (EtOAc). The organic layer was separated and washed with brine solution, dried over sodium sulfate, concentrated under reduced pressure. The residue was purified by flash chromatography on silica gel to afford **3a** (1.8 g, 89%). **^{1}H NMR** (500 MHz, $CDCl_3$) δ 7.15 (s, 1H), 5.05 (s, 1H), 4.14 (s, 1H), 3.73 (s, 3H), 3.31 (s, 2H), 3.27 (q, J = 6.8 Hz, 2H), 1.82 – 1.70 (m, 1H), 1.66 – 1.50 (m, 3H), 1.45 (s, 9H), 1.43 (s, 9H), 1.41 – 1.30 (m, 2H). **^{13}C NMR** (125 MHz, Chloroform-*d*) δ 172.0, 170.2, 165.0, 155.6, 82.0, 79.8, 53.9, 52.5, 41.0, 39.5, 32.8, 29.0, 28.5, 28.1, 22.7. **HRMS** (ESI$^+$): Calcd. for $C_{19}H_{35}N_2O_7^+$ [M+H]$^+$: 403.2439, found: 403.2438.

***N*^{6}-(3-methoxy-3-oxopropanoyl)-*L*-lysine (meMalK).** Compound **3a** (214 mg, 0.53 mmol) was treated with 4 M HCl in dioxane at 0 °C, and the reaction was stirred at room temperature. After the starting material was consumed completely which was detected by TLC, and the solvent was removed with a rotary evaporator. The residue was washed with diethyl ether, and the obtained precipitate was dried under high vacuum to afford the final product **meMalK** (quantitative yield). **^{1}H NMR** (500 MHz, DMSO-d_6) δ 8.43 (s, 1H), 8.42 (s, 1H), 8.23 (t, J = 5.6 Hz, 1H), 3.60 (s, 3H), 3.56 (s, 1H), 3.23 (s, 2H), 3.04 (q, J = 7.3, 6.8 Hz, 2H), 1.79 (h, J = 4.4, 3.4 Hz, 2H), 1.41 (dq, J = 13.1, 6.7, 5.8 Hz, 3H), 1.37 – 1.27 (m, 1H). **^{13}C NMR** (125 MHz, DMSO-d_6) δ 171.0, 168.5, 165.0, 51.8, 48.6, 42.3, 38.3, 29.6, 28.3, 21.7. **HRMS** (ESI$^+$): Calcd. for $C_{10}H_{19}N_2O_5^+$ [M+H]$^+$: 247.1288, found: 247.1286.

***tert*-butyl *N*^{2}-(*tert*-butoxycarbonyl)-*N*^{6}-(5-methoxy-5-oxopentanoyl)-*L*-lysinate (3b).**

Compound **2b** (750 µL, 6 mmol), HOBt (973 mg, 7.2 mmol), EDCI (1.4 g, 7.2 mmol) and DIPEA (4.37 mL, 25 mmol) were dissolved in dry THF. After 20 minutes, a solution of compound **1** (1.5 g, 5 mmol) in dry THF was added. The mixture was allowed to stir overnight. After the starting material was consumed completely which was detected by TLC, water was added to quench the reaction and extracted with ethyl acetate (EtOAc). The organic layer was separated and washed with brine solution, dried over sodium sulfate, concentrated under reduced pressure. The residue was purified by flash chromatography on silica gel to afford **3b** (480 mg, 22%). **^{1}H NMR** (500 MHz, $CDCl_3$) δ 5.89 (s, 1H), 5.09 (d, J = 8.3 Hz, 1H), 4.12 (d, J = 8.6 Hz, 1H), 3.66 (s, 3H), 3.22 (p, J = 7.6, 7.2 Hz, 2H), 2.36 (t, J = 7.1 Hz, 2H), 2.22 (t, J = 7.4 Hz, 2H), 1.95 (p, J = 7.3 Hz, 2H), 1.81 –

1.70 (m, 1H), 1.64 – 1.57 (m, 1H), 1.52 (tdd, J = 13.1, 11.3, 9.8, 5.5 Hz, 2H), 1.44 (s, 9H), 1.43 (s, 9H), 1.36 (tdt, J = 9.5, 6.1, 2.7 Hz, 2H). **^{13}C NMR** (125 MHz, Chloroform-*d*) δ 173.8, 172.4, 165.3, 155.7, 82.0, 79.8, 51.7, 39.4, 38.9, 35.5, 33.2, 32.8, 29.1, 28.5, 28.1, 22.7, 21.1. **HRMS** (ESI$^+$): Calcd. for $C_{21}H_{39}N_2O_7^+$ [M+H]$^+$: 431.2752, found: 431.2570.

***N*^{6}-(5-methoxy-5-oxopentanoyl)-*L*-lysine (meGluK).** Compound **3b** (252 mg, 0.59 mmol) was treated with 4 M HCl in dioxane at 0 °C, and the reaction was stirred at room temperature. After the starting material was consumed completely which was detected by TLC, and the solvent was removed with a rotary evaporator. The residue was washed with diethyl ether, and the obtained precipitate was dried under high vacuum to afford the final product **meGluK** (quantitative yield). **^{1}H NMR** (500 MHz, DMSO-*d*$_6$) δ 8.42 (s, 1H), 8.41 (s, 1H), 7.90 (t, J = 5.5 Hz, 1H), 3.58 (s, 3H), 3.56 (s, 1H), 3.00 (t, J = 6.1 Hz, 2H), 2.28 (t, J = 7.4 Hz, 2H), 2.08 (t, J = 7.4 Hz, 2H), 1.82 – 1.75 (m, 2H), 1.71 (q, J = 7.5 Hz, 2H), 1.39 (h, J = 6.9, 5.9 Hz, 3H), 1.34 – 1.25 (m, 1H). **^{13}C NMR** (125 MHz, DMSO-*d*$_6$) δ 173.1, 171.4, 171.0, 51.8, 51.3, 38.3, 34.3, 32.7, 29.6, 28.5, 21.7, 20.7. **HRMS** (ESI$^+$): Calcd. for $C_{21}H_{39}N_2O_7^+$ [M+H]$^+$:275.1601, found: 275.1599.

methyl (*S*)-2-((tert-butoxycarbonyl)amino)-3-(3-(pyrrolidin-1-yl)phenyl)propanoate (6a). Dehydrated DMF was added to zinc powder (3.25 g, 50.0 mmol) in a straight walled container under a nitrogen atmosphere. TMSCl (54.4 mg, 0.5 mmol) was added and the mixture was stirred at room temperature intensely for 30 minutes, then stopped stirring and allowed the zinc powder to settle. The supernatant was decanted under a flow of nitrogen. Then, dimethylformamide was added to the zinc powder, and the mixture was stirred for 2 minutes to allow the zinc powder to settle. The upper clear liquid was poured out again as before, this procedure was repeated twice more. 1,2-dibromoethane (751.4 mg, 4.0 mmol) was added to the mixture and stirred at 80 °C for an additional 30 minutes. After the mixture has cooled to room temperature, TMSCl (54.4 mg, 0.5 mmol) was added and stirred for 30 minutes. DMF solution of Boc-3-iodo-*L*-alaninemethyl ester (823 mg, 2.5 mmol) was added to the activated zinc powder and stirred vigorously. After the exothermic reaction was completed (controlled the temperature with an ice bath), stirring was continued for 30 minutes. The stirring was then stopped and the zinc powder was allowed to precipitate. The supernatant liquid was transferred to the mixture of 1-(3-bromophenyl)pyrrolidine (562.5 mg, 2.5 mmol), $Pd(OAc)_2$

(56.6 mg, 0.25 mmol), and S-Phos (102 mg, 0.25 mmol) via syringe. The reaction mixture was stirred at 40 °C for 4 h under a nitrogen atmosphere. After pouring into water, the mixture was extracted with ethyl acetate. The combined organic layer was washed with brine, dried over Na_2SO_4, and concentrated under reduced pressure. The residue was purified by flash chromatography on silica gel to afford the compound **6a** (470 mg, 54%). **^{1}H NMR** (500 MHz, DMSO-d_6) δ 7.22 (d, *J* = 8.1 Hz, 1H), 7.04 (t, *J* = 7.7 Hz, 1H), 6.44 (d, *J* = 7.4 Hz, 1H), 6.42 – 6.34 (m, 2H), 4.15 (ddd, *J* = 9.8, 8.0, 5.1 Hz, 1H), 3.61 (s, 3H), 3.23 – 3.14 (m, 4H), 2.90 (dd, *J* = 13.7, 5.1 Hz, 1H), 2.77 (dd, *J* = 13.7, 9.8 Hz, 1H), 1.95 – 1.90 (m, 4H), 1.33 (s, 9H). **^{13}C NMR** (125 MHz, DMSO-d_6) δ 172.8, 155.3, 147.7, 138.1, 128.8, 115.9, 112.3, 109.9, 78.2, 55.3, 51.8, 47.3, 36.9, 28.1, 25.0. **HRMS** (ESI$^+$): Calcd. for $C_{19}H_{29}N_2O_4^+$ [M+H]$^+$: 349.2122, found: 349.2122.

(*S*)-2-amino-3-(3-(pyrrolidin-1-yl)phenyl)propanoic acid (3THPF). Compound **6a** (470 mg, 1.35 mmol) was dissolved in 3 mL MeOH, and was added NaOH (160 mg, 4.0 mmol) in 3 mL H_2O. The mixture was stirred at room temperature for 2 h, the reaction was acidified with ice-cold 2M diluted hydrochloric acid and then washed with ice-cold diethyl ether. The aqueous phase was extracted with ice-cold ethyl acetate, then the combined organic layers was evaporated in vacuo to give a colorless oil with excellent yield without further purification. The compound was subsequently deprotected with 4M HCl/dioxane at 0 °C, delivering the amino acid **3THPF** (quantitative yield).**^{1}H NMR** (500 MHz, DMSO-d_6) δ 8.65 – 8.54 (m, 2H), 7.21 (t, J = 7.8 Hz, 1H), 6.78 (s, 2H), 4.10 (d, J = 5.7 Hz, 1H), 3.34 (d, J = 7.6 Hz, 4H), 3.13 (d, J = 6.0 Hz, 2H), 2.00 (p, J = 4.0, 3.5 Hz, 4H). **^{13}C NMR** (125 MHz, DMSO-d_6) δ 170.5, 137.5, 156.8, 144.6, 136.1, 129.3, 125.6, 120.2, 53.2, 47.8, 35.8, 24.6. **HRMS** (ESI$^+$): Calcd. for $C_{13}H_{19}N_2O_2^+$ [M+H]$^+$: 235.1441, found: 235.1438.

methyl (*S*)-2-((*tert*-butoxycarbonyl)amino)-3-(3-(dimethylamino)phenyl)propanoate (6b). The supernatant liquid was transferred to the mixture of 3-bromo-*N*,*N*-dimethylaniline (498 mg, 2.5 mmol), $Pd(OAc)_2$ (56.6 mg, 0.25 mmol), and S-Phos (102 mg, 0.25 mmol) via syringe. The reaction mixture was stirred at 40 °C for 4 h under a nitrogen atmosphere. After pouring into water, the mixture was extracted with ethyl acetate. The combined organic layer was washed with brine, dried over Na_2SO_4, and concentrated under reduced pressure. The residue was purified by flash

chromatography on silica gel to afford the compound **6b** (378 mg, 47%). **^{1}H NMR** (500 MHz, DMSO-d_6) δ 7.24 (d, J = 8.1 Hz, 1H), 7.06 (t, J = 7.8 Hz, 1H), 6.61 – 6.54 (m, 2H), 6.51 (d, J = 7.4 Hz, 1H), 4.15 (ddd, J = 9.9, 8.0, 5.0 Hz, 1H), 3.60 (s, 3H), 2.90 (dd, J = 13.7, 5.2 Hz, 1H), 2.86 (s, 6H), 2.77 (dd, J = 13.7, 9.9 Hz, 1H), 1.32 (s, 9H). **^{13}C NMR** (125 MHz, DMSO-d_6) δ 172.8, 155.4, 150.4, 138.0, 128.7, 117.0, 113.2, 110.7, 78.3, 55.3, 51.8, 40.2, 36.9, 28.2. **HRMS** (ESI$^+$): Calcd. for $C_{17}H_{27}N_2O_4^+$ [M+H]$^+$: 323.1965, found: 323.1962.

(*S*)-2-amino-3-(3-(dimethylamino)phenyl)propanoic acid (3DMAF). Compound **6b** (378 mg, 1.17 mmol) was dissolved in 3 mL MeOH, and was added NaOH (140 mg, 3.5 mmol) in 3 mL H_2O. The mixture was stirred at room temperature for 2 h, the reaction was acidified with ice-cold 2M diluted hydrochloric acid and then washed with ice-cold diethyl ether. The aqueous phase was extracted with ice-cold ethyl acetate, then the combine organic layer was evaporated in vacuo to give a colorless oil with excellent yield without further purification. And subsequently deprotected with 4M HCl/dioxane at 0 °C, delivering the amino acid **3DMAF** (quantitative yield). **^{1}H NMR** (500 MHz, DMSO-d_6) δ 8.65 (s, 2H), 7.64 (d, J = 64.3 Hz, 2H), 7.49 – 7.18 (m, 2H), 4.19 (d, J = 5.8 Hz, 1H), 3.22 (d, J = 6.3 Hz, 2H), 3.06 (s, 6H). **^{13}C NMR** (125 MHz, DMSO-d_6) δ 170.5, 137.5, 130.3, 63.3, 53.4, 49.0, 35.9. **HRMS** (ESI$^+$): Calcd. for $C_{11}H_{17}N_2O_2^+$ [M+H]$^+$: 209.1285, found: 209.1283.

Assessment of amber suppression efficiency by GFP reporter assay in *E. coli*

For screening the active mutants for meGluK and meMalK, the plasmids carrying PylRS mutants reported to recognize lysine derivatives in our lab were chosen and co-transformed with plasmid carrying GFP190TAG-PylT into DH10B competent cells. For screening the active mutants for the designed ncAA with stronger cation-π binding energy, the plasmids carrying chPheRS mutants in our lab were chosen and co-transformed with plasmid carrying GFP190TAG-chPheT into DH10B competent cells. The clones were picked and cultured in the LB medium containing 50 μg/mL kanamycin and 100 μg/mL ampicillin. The expression was induced in the presence or absence of the corresponding ncAA (2 mM). After induction, 0.75 mL cell cultures were collected by centrifugation and then lysed with 150 μL BugBuster Protein Extraction Reagent (Millipore) for 20

min at room temperature. The supernatant of the lysate (100 μL) was transferred to a 96-well cell culture plate (Costar). Fluorescence and OD600 were measured by Bio Tek Synergy NEO2.

Protein expression, purification and LC–MS analysis

Protein expression and purification were carried out as described previously.[3] Purified proteins were sent for LC-MS analysis. The yield of purified protein was obtained by measuring the absorbance at 280 nm (395 nm for GFP variants) using a spectrometer. The molecular mass of the protein was predicted using the ExPASy Compute pI/Mw tool and chromophore maturation in GFP was also considered in the calculation.

For LC-MS analysis, SCIEX Triple TOF 6600 MS System with an electrospray ionization source was used. The procedures for separating and desalting were performed as before.[3] Mass spectral deconvolution was performed using SCIEX OS-Q software (v.2.0, SCIEX Corporation).

FACS analysis of amber suppression efficiency in mammalian cells

For FACS analysis in live cells, HEK 293T cells were seeded in a 24-well plate and grown to 60-80% confluency for transfection. Plasmid pEGFP-mCherry-T2A-EGFP190TAG was co-transfected with pCMV plasmid bearing corresponding aaRS/tRNA into cells via Lip2000 transfection reagent according to the manufacturer's protocol, with or without the corresponding ncAAs. At 48 h post-transfection, cells were trypsinized and neutralized by the complete medium before centrifugation. Cells were centrifuged at 1,400g for 3 min, washed, and resuspended in PBS for FACS analysis. The detailed gating method was described previously.[3]

Calculating Cation-π Binding Energies

All calculations were performed with Gaussian 16.[4] Molecular geometries were optimized at the M06-2X/6-31G(d) level of theory.[5] Vibrational frequencies were evaluated at the same level to verify the optimized structure as minima. For electrostatic potential maps, calculations were performed at the B3LYP/6-311+G(d,p) level of theory.[6] Cation–π binding energies (CπBE) were calculated for the aromatic side chain with the sodium ion using single-point energy calculations at the M06-2X/6-311+G(d,p) level of theory. The computed CπBE was defined as the energy difference between the complex of electron-rich substituted aromatic side chain with the sodium ion

($E_{complex}$) and independent electron-rich substituted aromatic side chain (E_{π}) and sodium ion (E_{+}): $C\pi BE = E_{complex} - (E_{\pi} - E_{+})$.

Microscale thermophoresis (MST)

Protein-peptide interactions were analyzed by microscale thermophoresis (MST). $H3_{1-14}K4me3$ peptide was synthesized and labeled with fluorescein isothiocyanate (FITC). All samples were desalted with MST buffer (20 mM Tris-HCl, 50 mM NaCl, 1 mM DTT, 0.05% Tween-20, pH 7.5). MST was performed with a NT.115 Monolith instrument (Nano Temper Technologies, Munich, Germany) using a Blue LED for excitation in three independent replicates at 25 °C. The instrument settings were: 20% blue LED excitation power, 40% IR laser power. The measurements were performed in standard glass capillaries (Nano Temper Technologies, cat#MO-K022). All dissociation constants (Kd) were calculated to a binding model assuming a 1:1 stoichiometry per binding partner by NT Analysis Software.

Code availability

The data and code for building the model of ncAA screener was uploaded onto Zenodo (https://zenodo.org/records/14043541)[6-7].

Supplementary Table 1. The aromatic boxes of lysine methylation reader domains. The aromatic cage of human proteins with Uniprot-annotated Chromo, PHD, PWWP, Tudor, MBT, CW, SPIN and BAH domains were collected and classified by the number of Tyr and Phe residues in their aromatic boxes.

Domain	Number of Tyr or Phe	Reader
Chromo	0	CHD3 (Chromo 1), CHD4 (Chromo 1), CHD5 (Chromo 1), CHD1 (Chromo 2), CHD2 (Chromo 2), CHD3 (Chromo 2), CHD4 (Chromo 2), CHD5 (Chromo 2), CHD6 (Chromo 2), CHD7 (Chromo 2), CHD8 (Chromo 2), CHD9 (Chromo 2)
	1	CBX2, CBX4, CBX6, CBX7, CBX8, CDYL1, CHD1 (Chromo 1), CHD2 (Chromo 1), SMARCC1, SMARCC2, SUV39H2
	2	ARID4A, ARID4B, CBX1, CBX3, CBX5, CDY1, CDY2, CDYL2, CHD6 (Chromo 1), CHD7 (Chromo 1), CHD8 (Chromo 1), CHD9 (Chromo 1), KAT5, KAT8, MPP8, MRG15, MSL3, SUV39H1
PHD	0	CHD3, CHD4, KDM5A, KMT2E, SET (Yeast), TAF3
	1	ASH1L, DIDO1, ING1, ING2, ING3, ING4, ING5, KMT2B, KMT2C, KMT2D, KMT2E, PYGO1, RAG2, Yng1 (Yeast)
	2	KDM2A, KDM2B, KDM4A, KDM4B, KDM4C, KMT2A, MTF2, PHF2, PHF8
	3	BPTF, KAT6A
PWWP	0	MBD5, NSD1
	1	ARID4A, ARID4B, DNMT3A, DNMT3B, PWWP2B, ZCWPW2
	2	GLYR1, HDGF, HDGFL1, HDGFRP2, HDGFRP3, MSH6, NSD2, NSD3, PSIP1, PWWP3A, ZMYND8, ZMYND11
	3	BRPF1, BRPF2, BRPF3, ZCWPW1
Tudor	1	SETDB1, TDRD12
	2	KDM4A, KDM4B, LBR, MTF2, TDRD6, TDRD7,
	3	PHF1, PHF19, PHF20, SGF29, SMN1, SMNDC1, SPIN1, TDRD1, TDRD2, TDRD10, TDRKH, TP53BP1, UHRF1
	4	SND1, STK31, TDRD4, TDRD9
MBT	2	L3MBTL, L3MBTL2, MBTD1, SCMH1, SCML2, SFMBT1, SFMBT2
	3	L3MBTL3
SPIN	3	SPIN1, SPIN3, SPIN4, SPIN2A, SPIN2B
BAH	2	BAHD1, hORC1

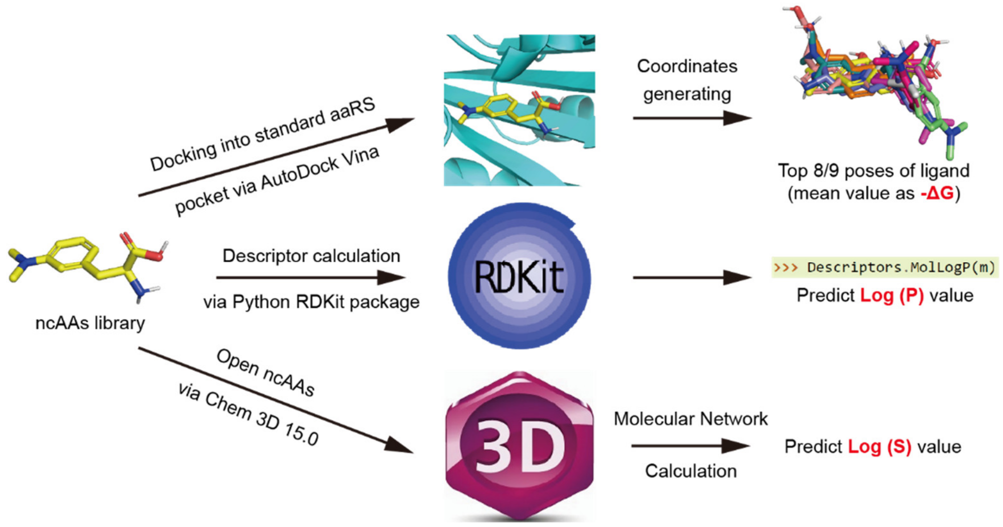

Supplementary Figure 1. The workflow for the calculation of chemical properties of ncAA.

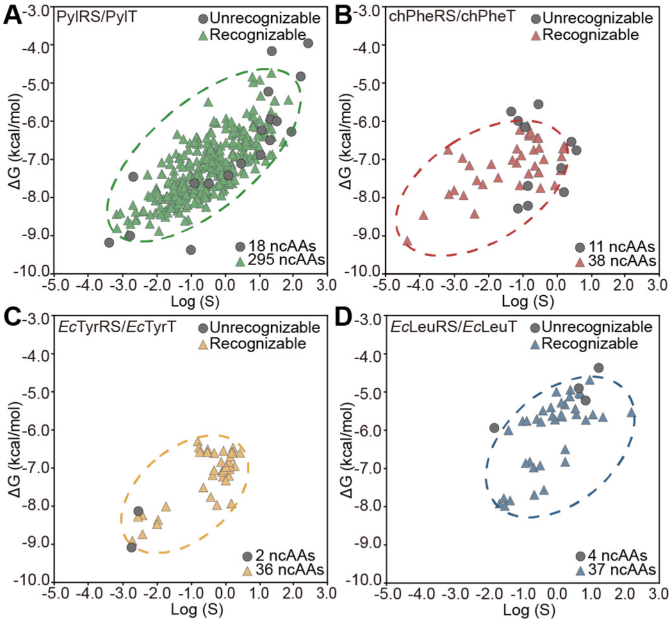

Supplementary Figure 2. The virtual ncAA screeners based on Log (S) and ΔG values. (A) Pyl system. **(B)** chPhe system. **(C)** *Ec*Tyr system. **(D)** *Ec*Leu system.

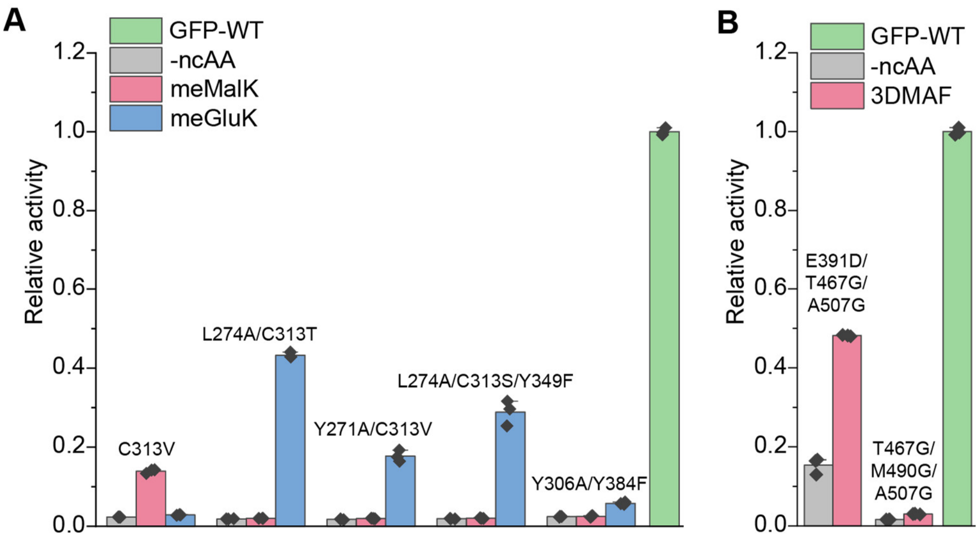

Supplementary Figure 3. The amber suppression efficiency of aaRSmutants for ncAAs. The amber suppression efficiency of PylRS mutants **(A)** and chPheRS mutants **(B)** were tested by a GFP reporter assay with or without the addition of indicated ncAA. The PylRS mutants of C313V and L274A/C313T were dubbed as meMalKRS and meGluKRS respectively. The chPheRS mutant of E391D/T467G/A507G was chPheRS-3 reported before. Error bars represent ± standard error of the mean (n = 3).

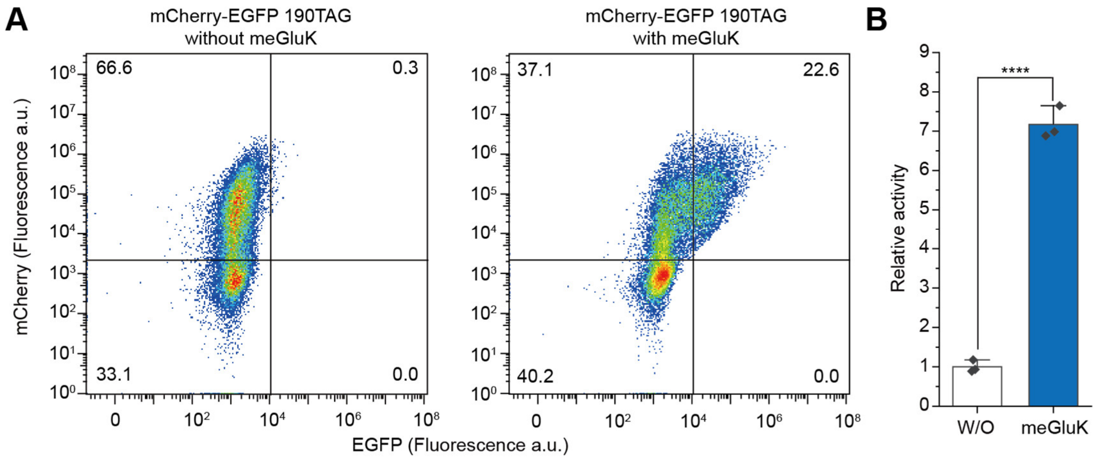

Supplementary Figure 4. The amber suppression efficiency of meGluK system in mammalian cells. (A) Flow cytometry analysis of the amber suppression efficiency of mCherry-EGFP 190TAG by the meGluK translation system with or without the addition of meGluK in HEK 293T cells. **(B)** The relative activity of meGluK incorporation obtained from panel (A). The activity was normalized by the group in the absence of meGluK set as 1. Error bars represent the ± standard error of the mean from three biologically independent experiments. Statistical significance was quantified with t-test(****$p < 0.0001$).

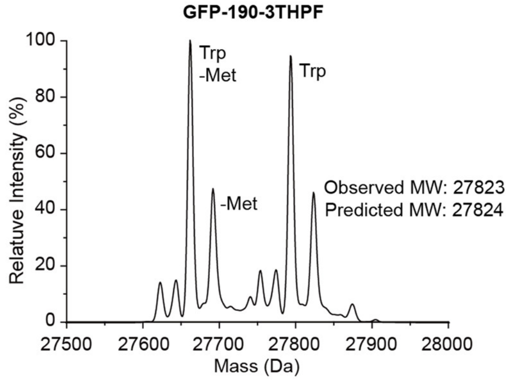

Supplementary Figure 5 The mass spectrometry characterization of the fidelity of 3THPF incorporation into GFP by chPheRS-3. The expected molecular mass (MW) value of GFP incorporated with 3THPF was 27824 Da, and the observed MW value was 27823 Da. The peak with N-Met cleavage was also detected. However, the peaks with incorporated with Trp were also detected, indicating the relatively low fidelity of 3THPF incorporation.

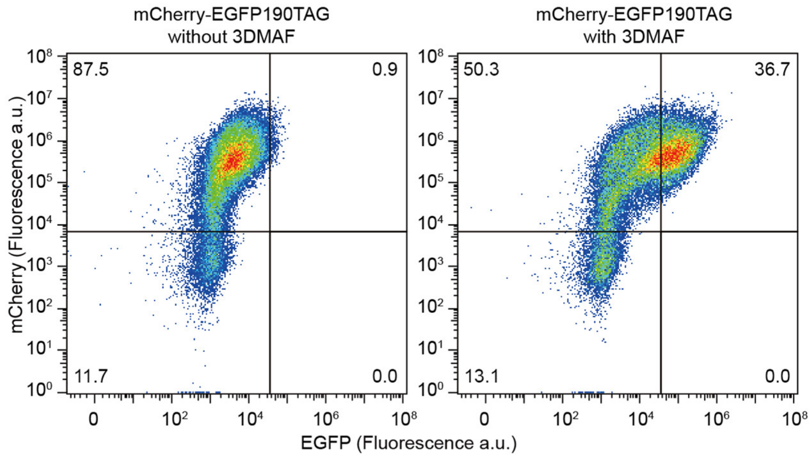

Supplementary Figure 6. The amber suppression efficiency of 3DMAF system in mammalian cells. The reporter of mCherry-T2A-EGFP 190TAG was co-transfected with chPheRS-3/chPheT in presence or absence of 3DMAF.

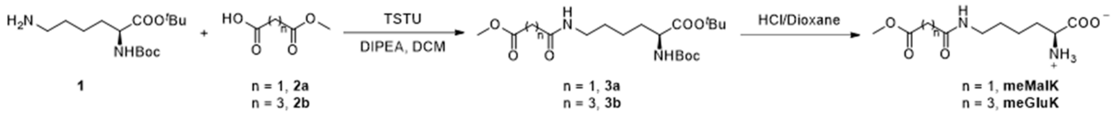

Supplementary Figure 7. Chemical synthesis of meMalK and meGluK.

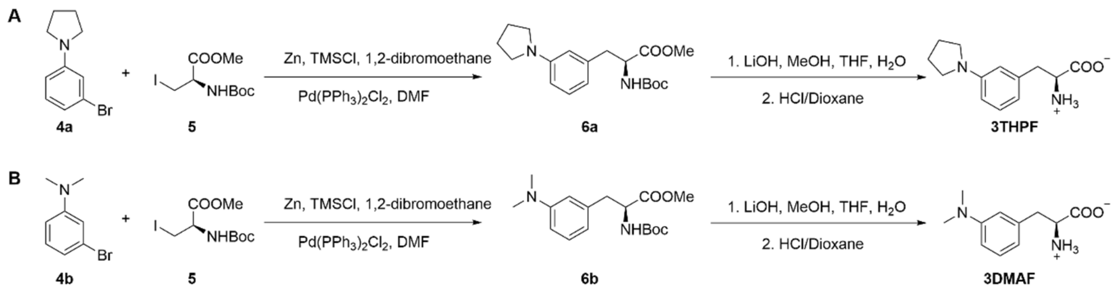

Supplementary Figure 8. Chemical synthesis of 3THPF (A) and 3DMAF (B).

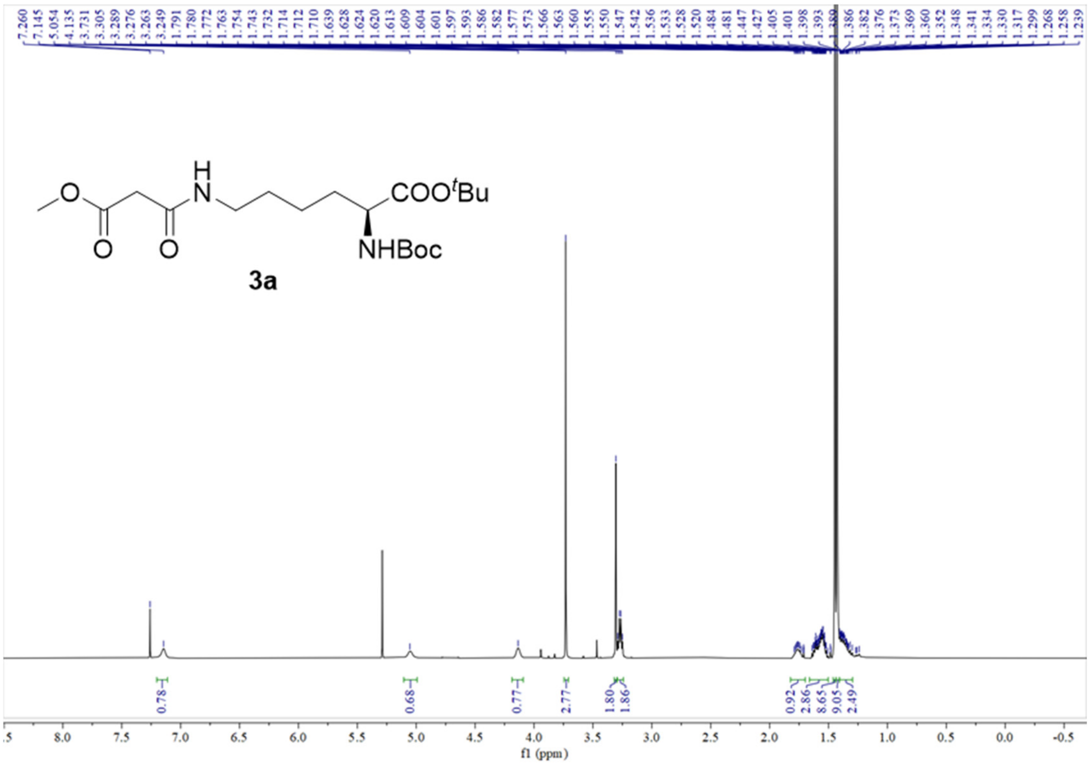

Supplementary Figure 9. ^{1}H NMR of compound 3a.

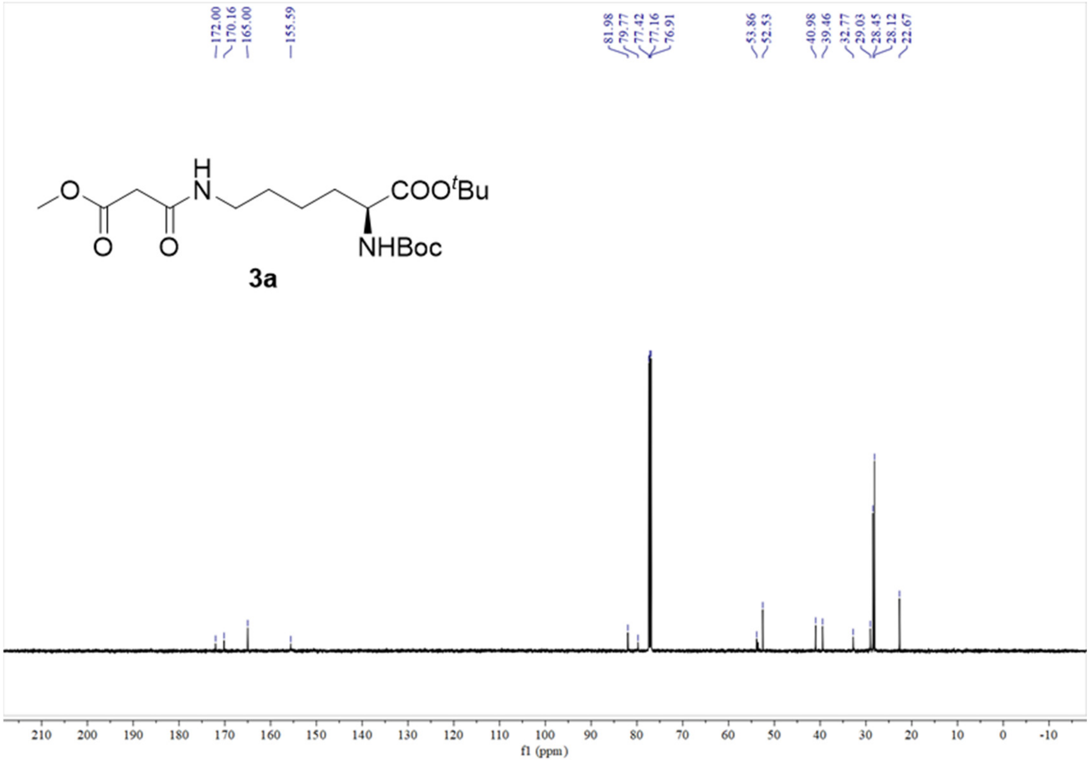

Supplementary Figure 10. ^{13}C NMR of compound 3a.

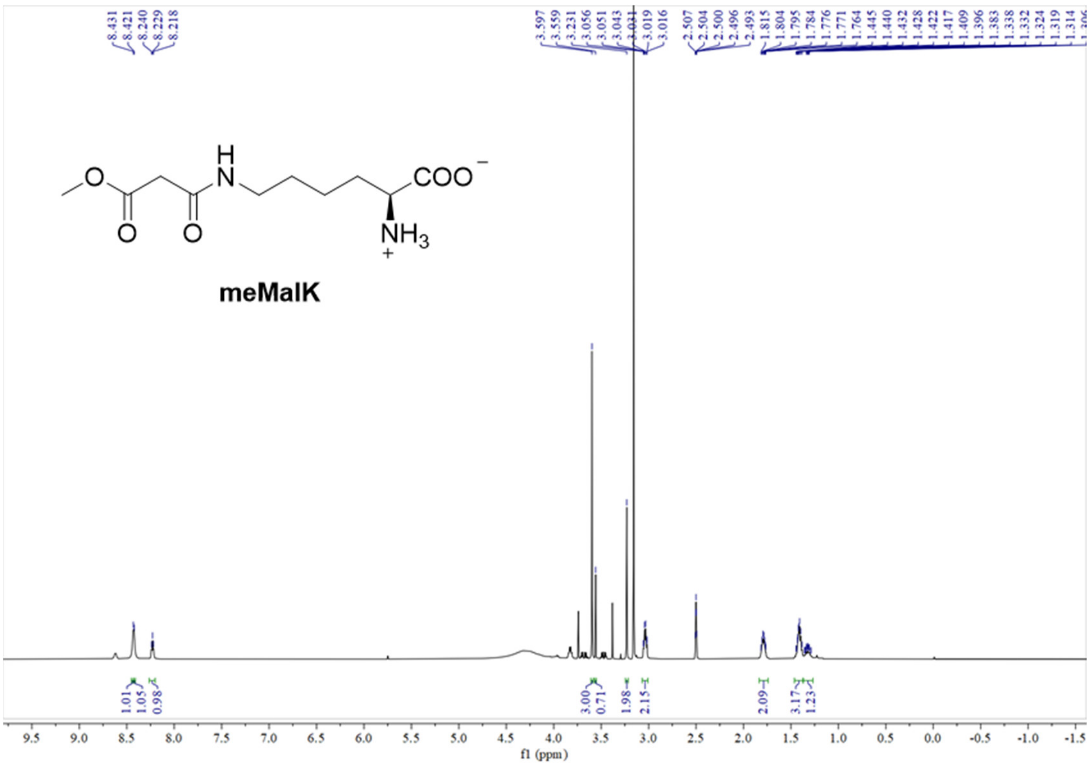

Supplementary Figure 11. ^{1}H NMR of compound meMalK.

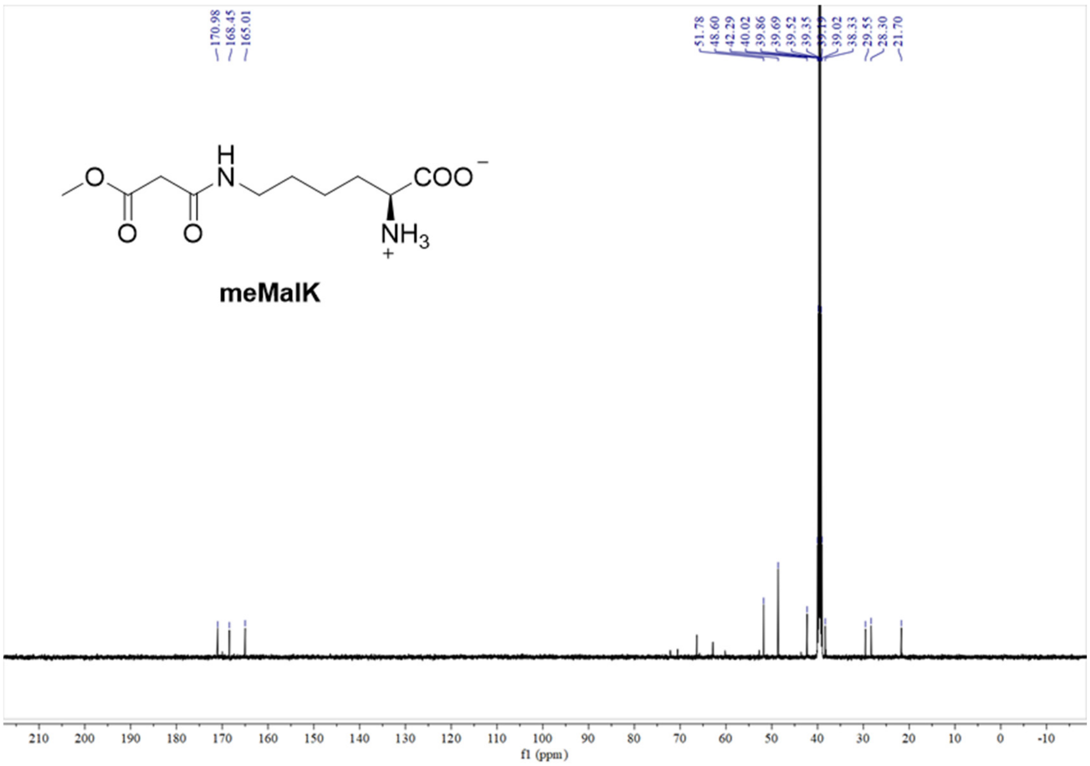

Supplementary Figure 12. ^{13}C NMR of compound meMalK.

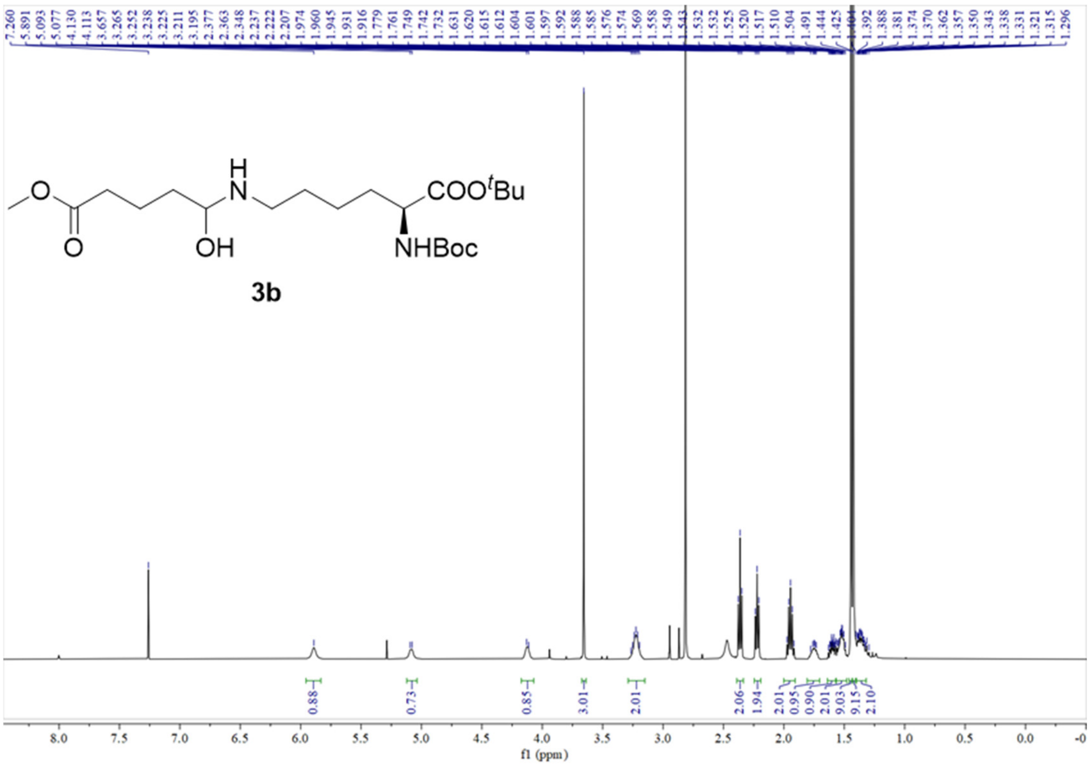

Supplementary Figure 13. ^{1}H NMR of compound 3b.

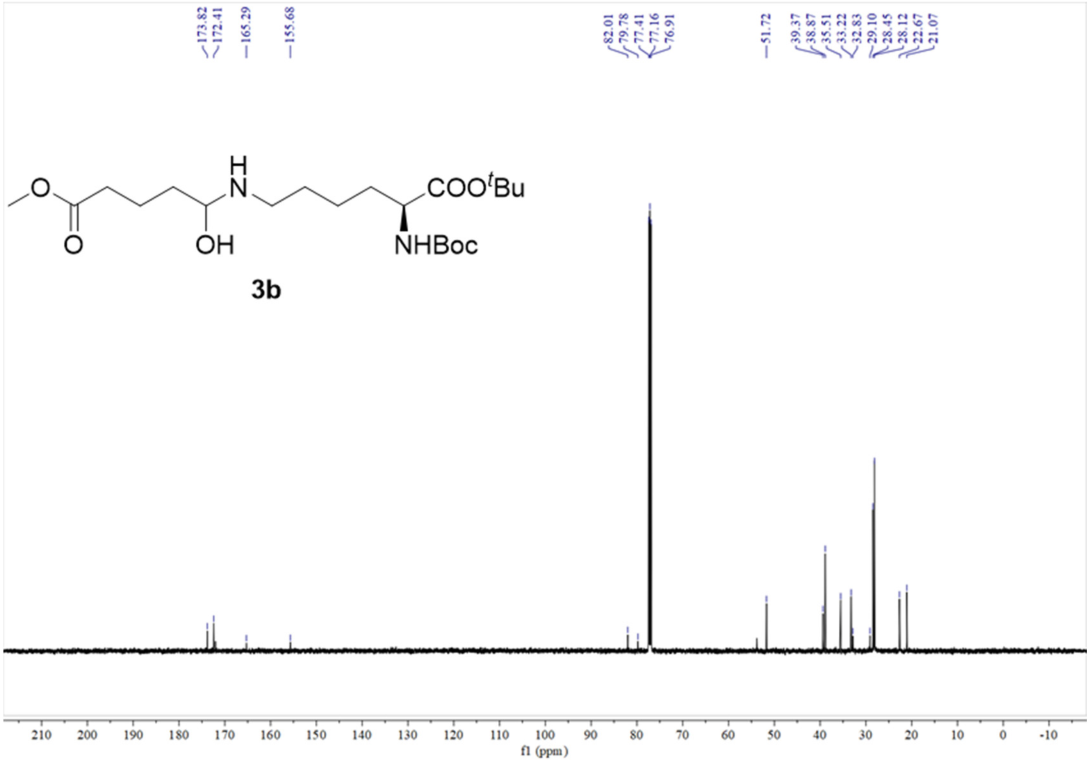

Supplementary Figure 14. ^{13}C NMR of compound 3b.

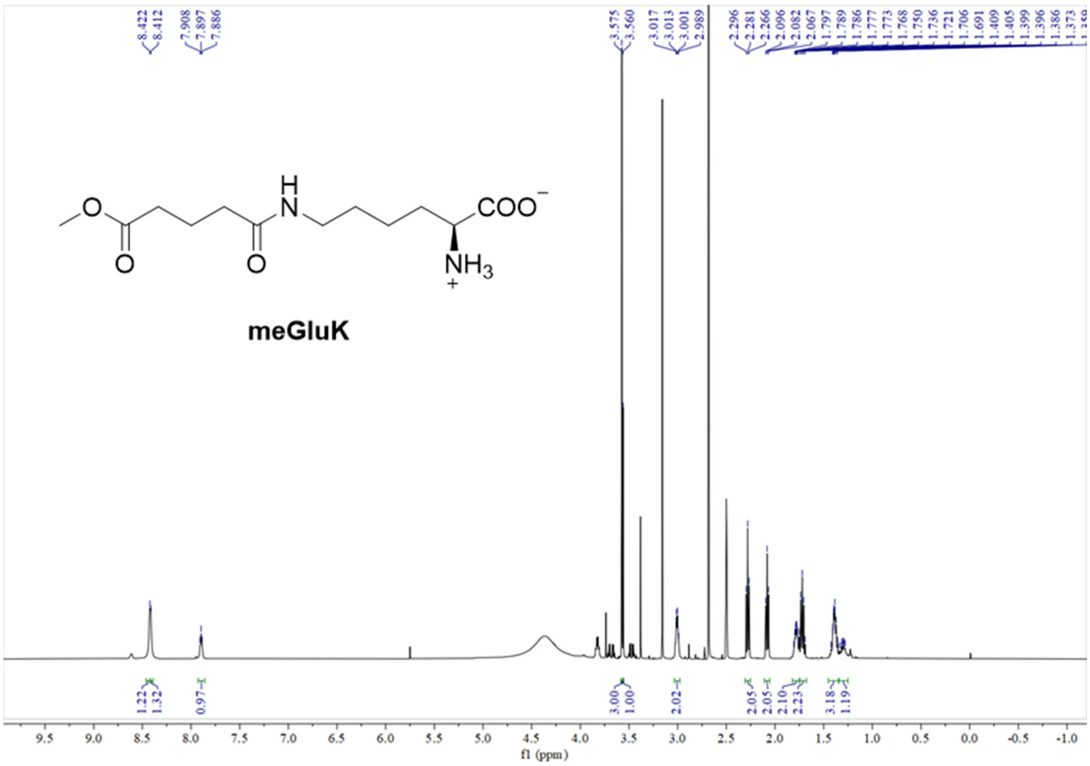

Supplementary Figure 15. ^{1}H **NMR of compound meGluK.**

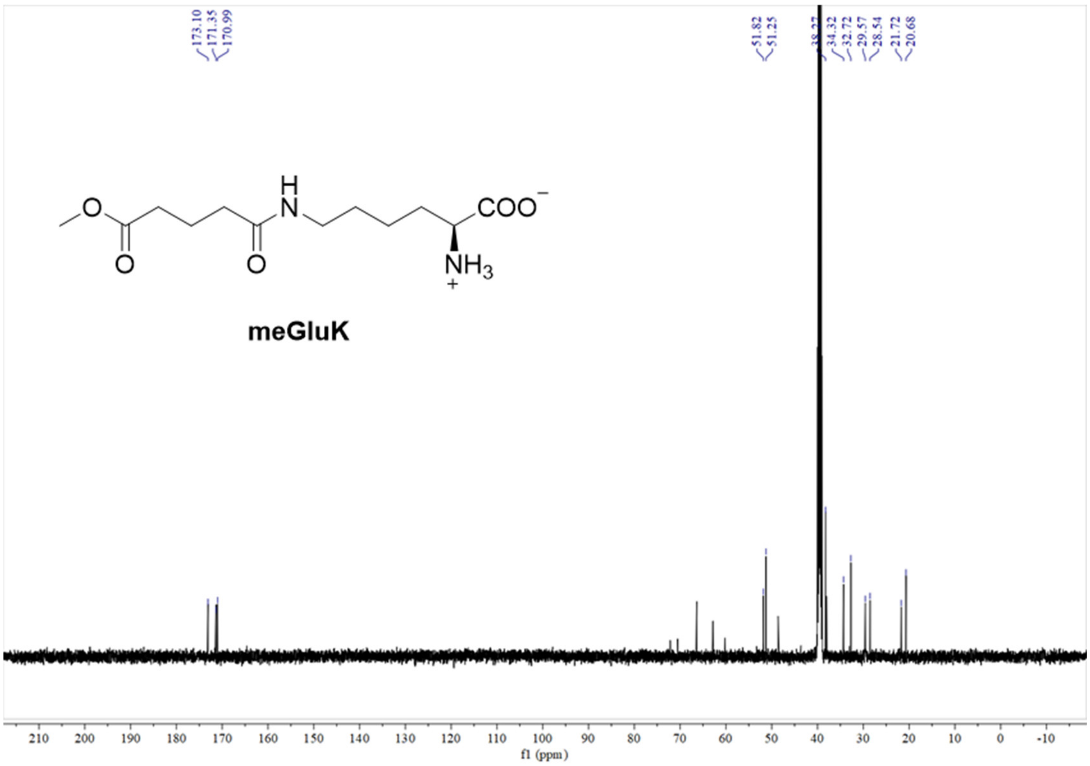

Supplementary Figure 16. ^{13}C **NMR of compound meGluK.**

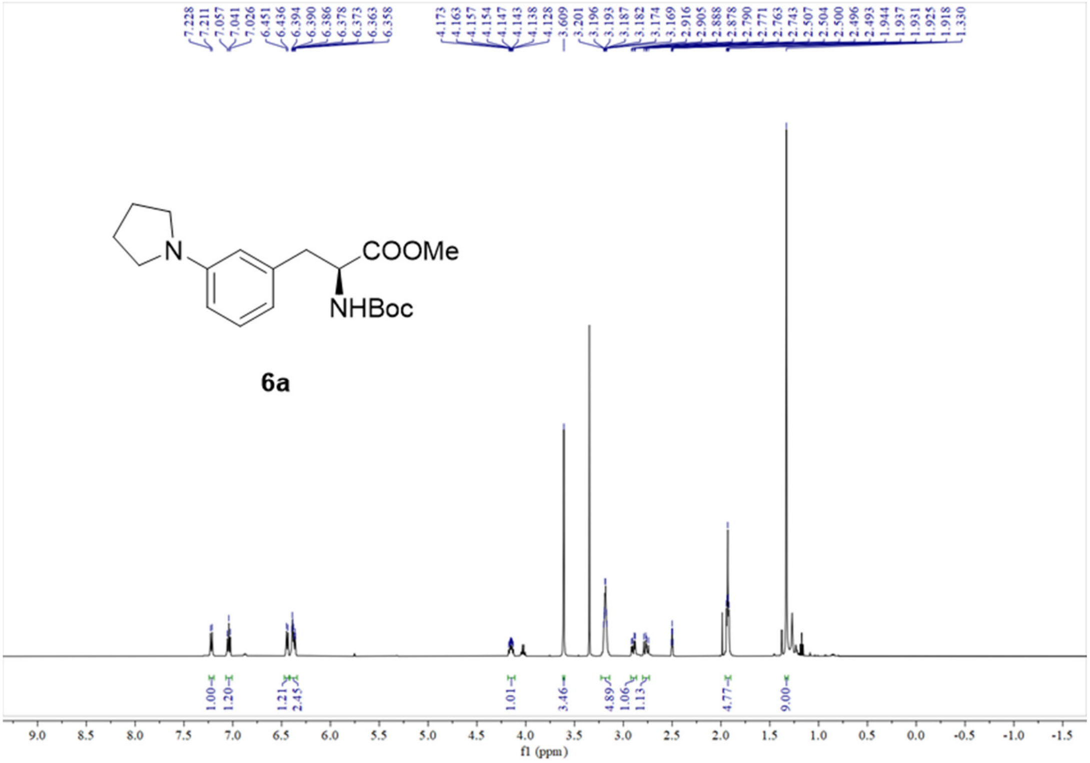

Supplementary Figure 17. ^{1}H NMR of compound 6a.

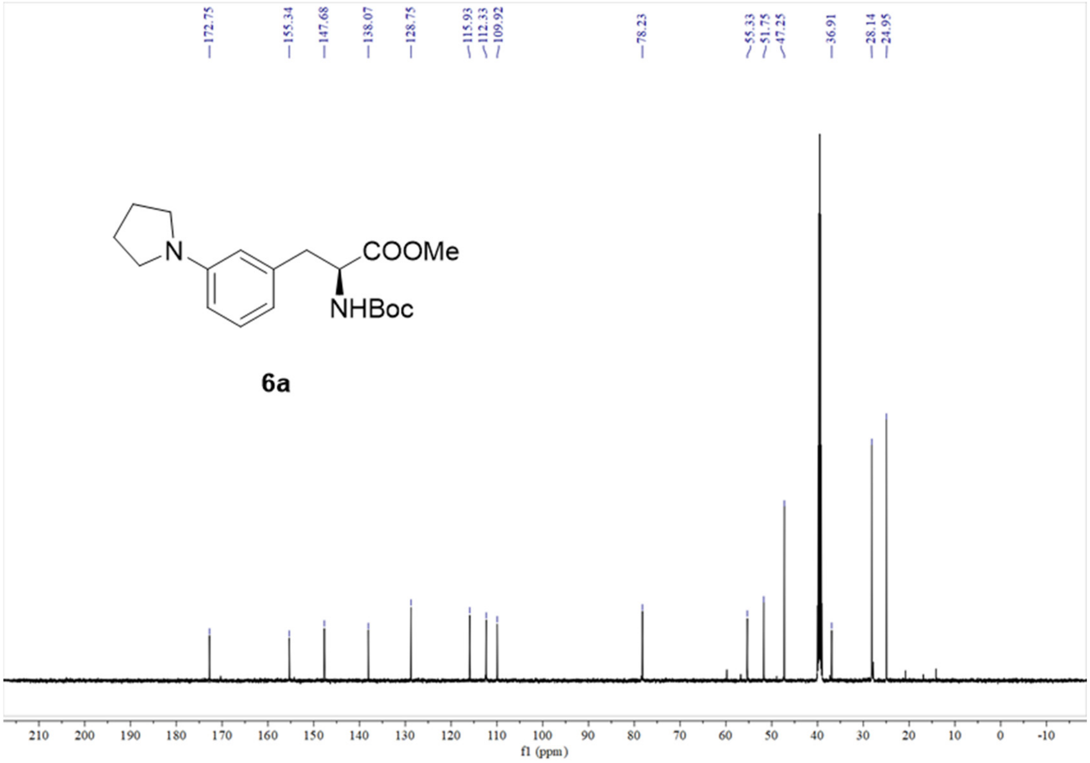

Supplementary Figure 18. ^{13}C NMR of compound 6a.

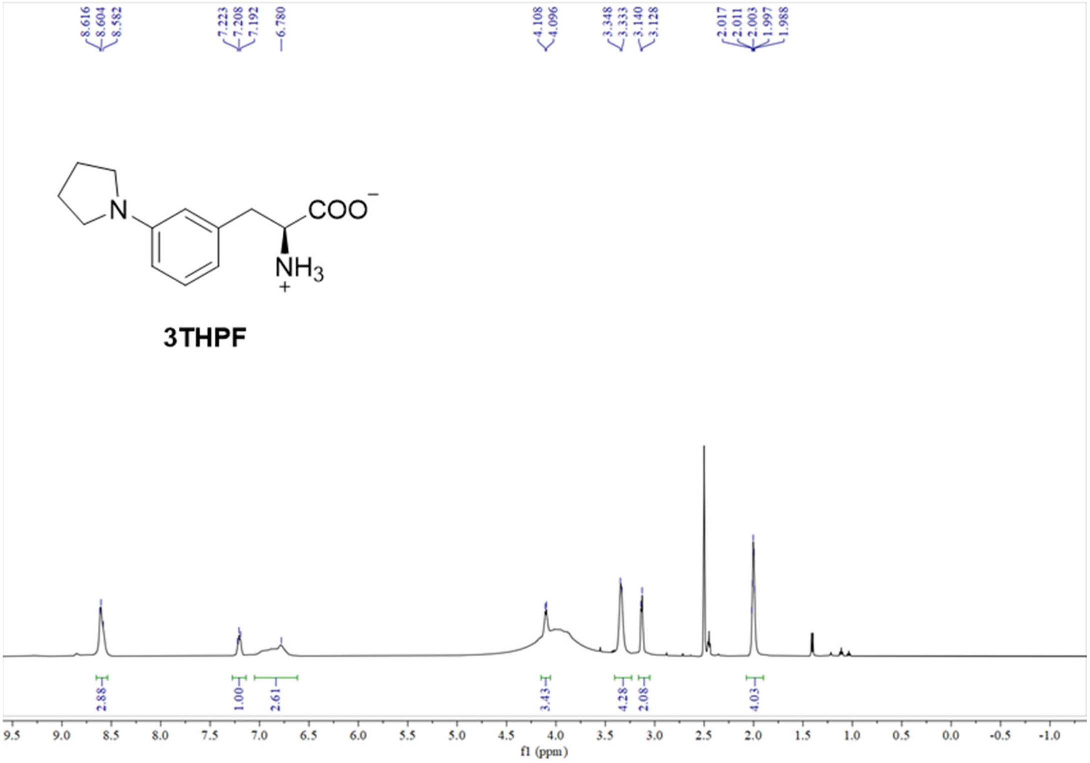

Supplementary Figure 19. ^{1}H NMR of compound 3THPF.

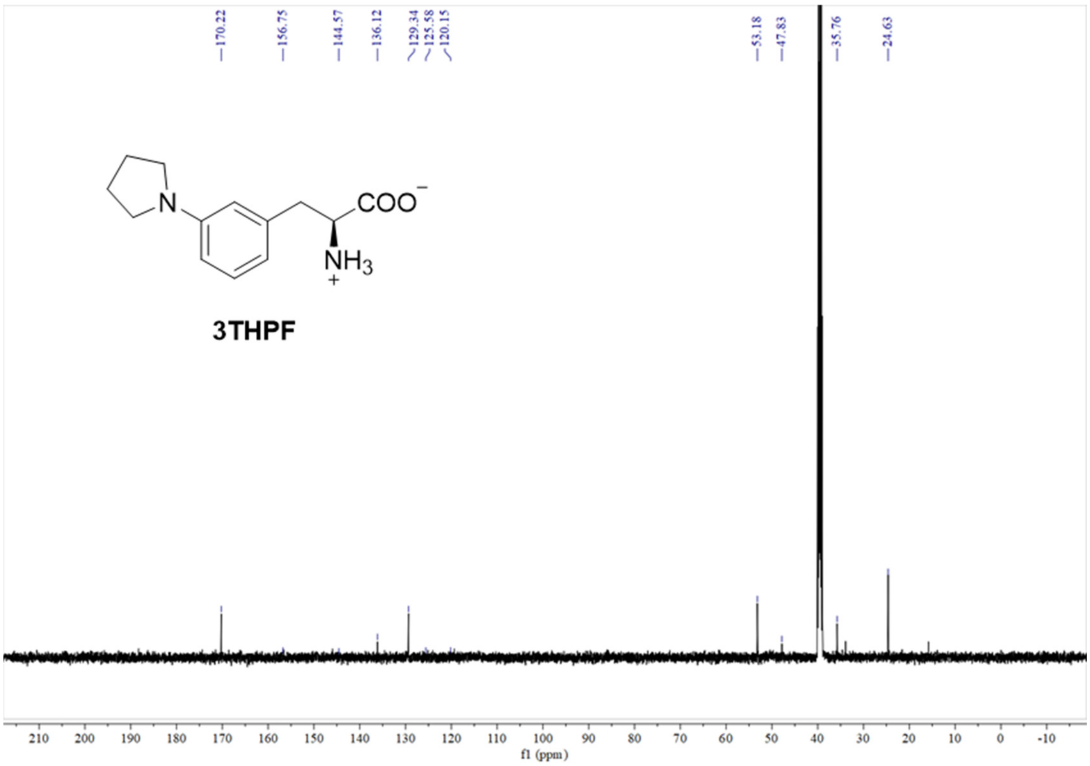

Supplementary Figure 20. ^{13}C NMR of compound 3THPF.

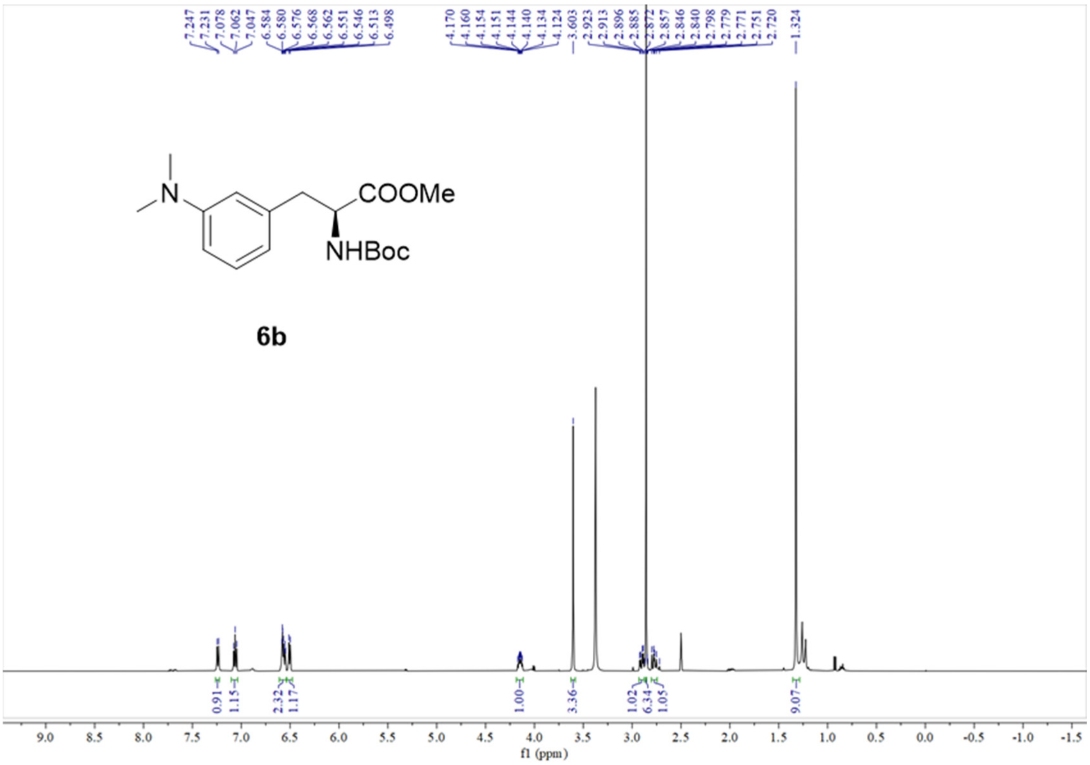

Supplementary Figure 21. ^{1}H NMR of compound 6b.

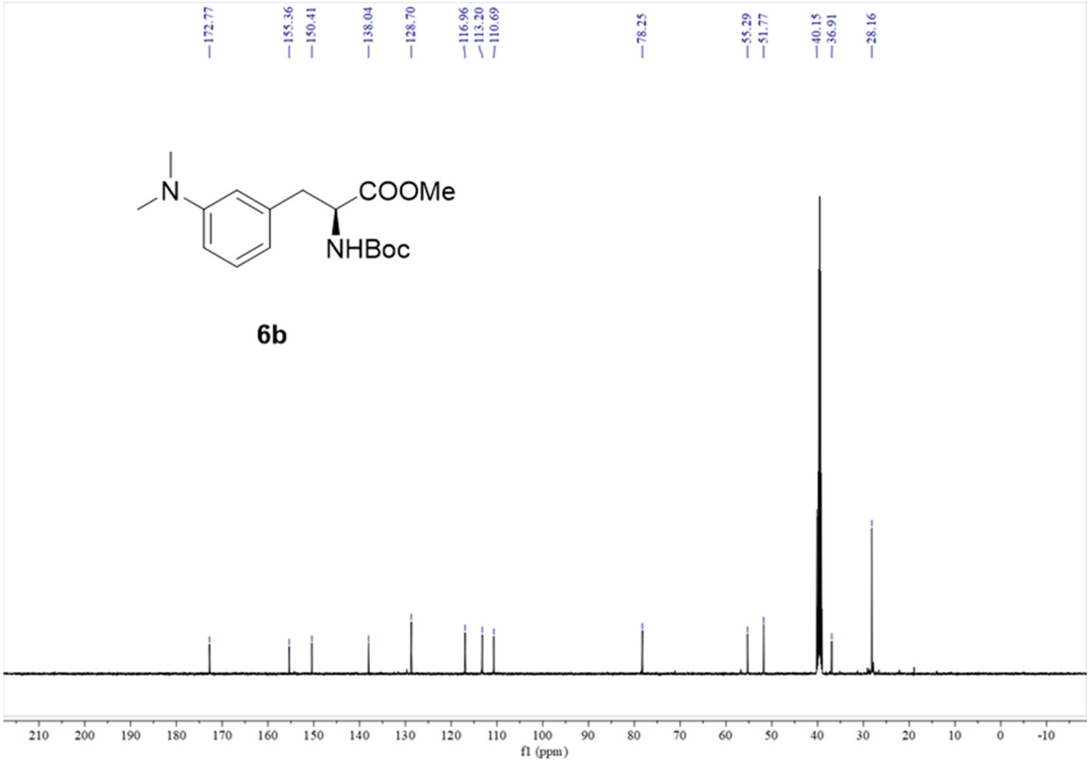

Supplementary Figure 22. ^{13}C NMR of compound 6b.

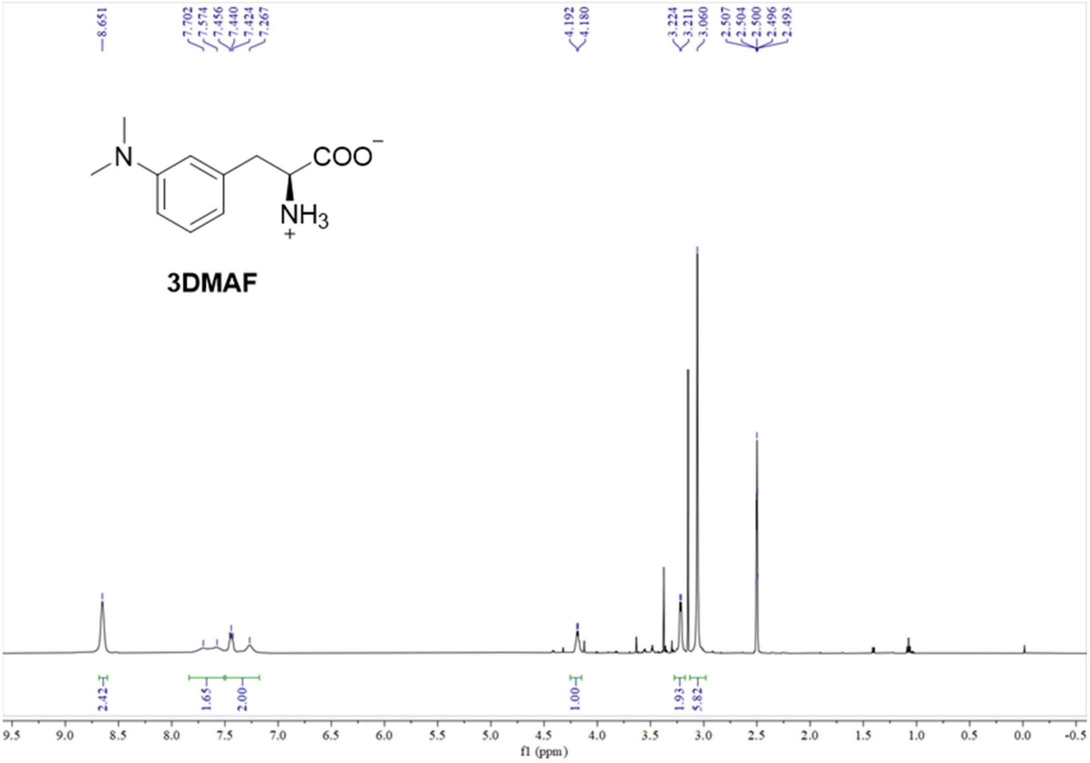

Supplementary Figure 23. ^{1}H **NMR of compound 3DMAF.**

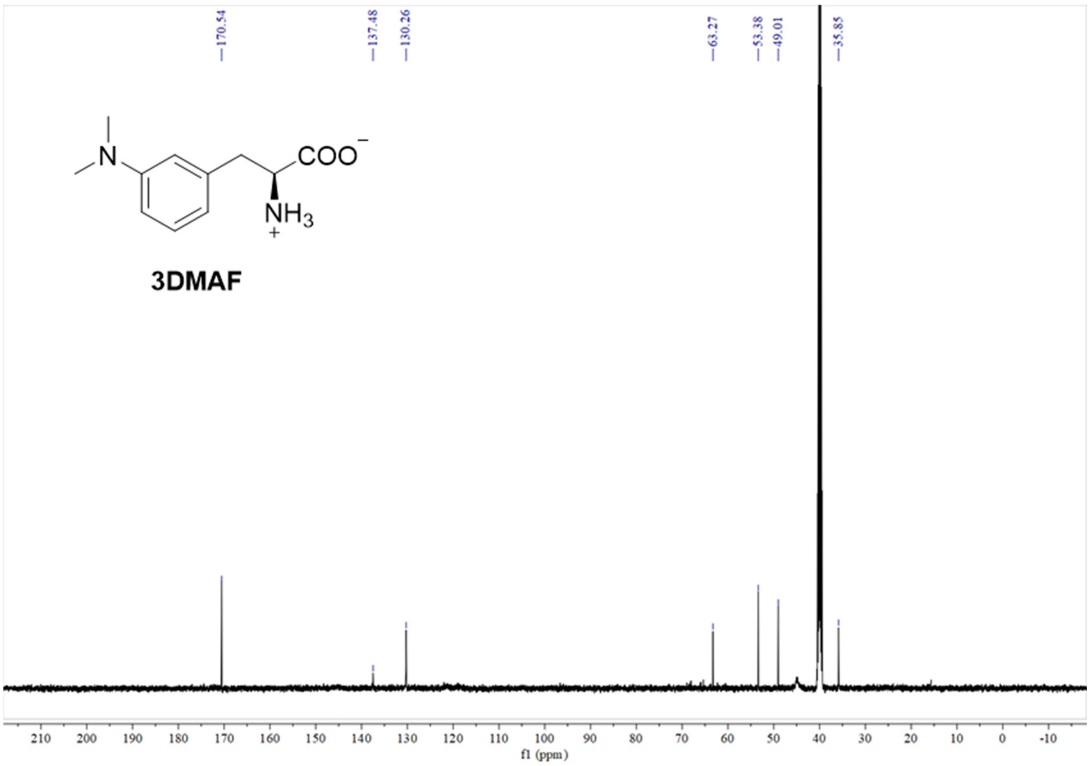

Supplementary Figure 24. ^{13}C **NMR of compound 3DMAF.**

➢ References

1. Eberhardt, J.; Santos-Martins, D.; Tillack, A. F.; Forli, S., AutoDock Vina 1.2.0: New Docking Methods, Expanded Force Field, and Python Bindings. *J Chem Inf Model* **2021,** *61*, 3891-3898.
2. Morris, G. M.; Huey, R.; Lindstrom, W.; Sanner, M. F.; Belew, R. K.; Goodsell, D. S.; Olson, A. J., AutoDock4 and AutoDockTools4: Automated docking with selective receptor flexibility. *J. Comput. Chem.* **2009,** *30*, 2785-91.
3. Ding, W.; Liu, C.; Chen, Y.; Gu, J.; Fang, C.; Hu, L.; Zhang, L.; Yuan, Y.; Feng, X. H.; Lin, S., Computational design and genetic incorporation of lipidation mimics in living cells. *Nat. Chem. Biol.* **2024,** *20*, 42-51.
4. Frisch, M. J.; Trucks, G. W.; Schlegel, H. B.; Scuseria, G. E.; Robb, M. A.; Cheeseman, J. R.; Scalmani, G.; Barone, V.; Petersson, G. A.; Nakatsuji, H.; Li, X.; Caricato, M.; Marenich, A. V.; Bloino, J.; Janesko, B. G.; Gomperts, R.; Mennucci, B.; Hratchian, H. P.; Ortiz, J. V.; Izmaylov, A. F.; Sonnenberg, J. L.; Williams; Ding, F.; Lipparini, F.; Egidi, F.; Goings, J.; Peng, B.; Petrone, A.; Henderson, T.; Ranasinghe, D.; Zakrzewski, V. G.; Gao, J.; Rega, N.; Zheng, G.; Liang, W.; Hada, M.; Ehara, M.; Toyota, K.; Fukuda, R.; Hasegawa, J.; Ishida, M.; Nakajima, T.; Honda, Y.; Kitao, O.; Nakai, H.; Vreven, T.; Throssell, K.; Montgomery Jr, J. A.; Peralta, J. E.; Ogliaro, F.; Bearpark, M. J.; Heyd, J. J.; Brothers, E. N.; Kudin, K. N.; Staroverov, V. N.; Keith, T. A.; Kobayashi, R.; Normand, J.; Raghavachari, K.; Rendell, A. P.; Burant, J. C.; Iyengar, S. S.; Tomasi, J.; Cossi, M.; Millam, J. M.; Klene, M.; Adamo, C.; Cammi, R.; Ochterski, J. W.; Martin, R. L.; Morokuma, K.; Farkas, O.; Foresman, J. B.; Fox, D. J. *Gaussian 16 Rev. C.01*, Wallingford CT, 2016.
5. Zhao, Y.; Truhlar, D. G., The M06 suite of density functionals for main group thermochemistry, thermochemical kinetics, noncovalent interactions, excited states, and transition elements: two new functionals and systematic testing of four M06-class functionals and 12 other functionals. *Theor. Chem. Acc.* **2007,** *120*, 215-241.
6. Stephens, P. J. D., F. J.; Chabalowski, C. F.; Frisch, M. J., Ab-initio calculation of vibrational absorption and circular-dichroism spectra using density-functional force-fields. *J. Phys. Chem.* **1994,** *98*, 11623-11627.
7. Fang, C., Code of 'Computationally assisted noncanonical amino acid incorporation'. *Zenodo* **2024**, https://doi.org/10.5281/zenodo.14043541.
