# Supplementary material for: Computationally Assisted Noncanonical Amino Acid Incorporation
Source: ACS Cent Sci. 2024 Dec 16;11(1):84–90. doi: 10.1021/acscentsci.4c01544 (PMC11758377; doi:10.1021/acscentsci.4c01544)
Supplement: Supplementary file 3 — oc4c01544_si_003.pdf [file oc4c01544_si_003.pdf]

oc-2024-01544c.R1

Name: Peer Review Information for "Computationally assisted noncanonical amino acid incorporation"

First Round of Reviewer Comments

Reviewer: 1

Comments to the Author

This manuscript presents a computational model to evaluate the recognition potential of candidate ncAAs by four widely used orthogonal aaRSs. Overall, the work is original, and the model is valuable. It can be published with minor revisions.

1. It is well known that PylRS prefers amino acids with hydrophobic side chains. Therefore, it is not surprising that PylRS does not recognize the negatively charged MalK and GluK, even without the computational model. These examples do not effectively support the authors' claim.

2. For Figures 3C and 4C, a control—such as wild-type or a reported mutant—should be included for comparison.

3. Intuitively, a computational method that aids in the design of aaRSs to recognize new ncAAs would be more valuable than a program that simply advises against proceeding due to potential failures with existing and commonly used protein engineering approaches.

Nevertheless, it would be beneficial if the authors could outline how they plan to make the model accessible to other researchers.

Reviewer: 2

Comments to the Author

Noncanonical amino acids (ncAAs) mutagenesis is an invaluable tool in protein-based research and therapeutics. One of the current limitations is the structural diversity of sidechains that can be genetically encoded by engineered aminoacyl tRNA synthetase (aaRS). To this end, this manuscript by Fang, et al. developed a virtual screener to determine the recognition potential of ncAAs by analysis and modeling of the side chain structures. Well, most other groups are struggling with computationally designed enzymes to recognize a synthesized ncAAs, this work provide an alternative strategy to virtually screen the recognition potential prior to chemical synthesis. Overall, I find that the manuscript very interesting and would be suitable for publication in ACS Central Science. However, some minor concerns need to be address first.

1. Please define the detailed scientific rationale for parameter selection. Currently, the development of the virtual screening model is primarily based on the permeability, solubility, and binding free energy (ΔG) of ncAAs with aaRS. However, the scientific rationale behind the selection of these parameters is not thoroughly explained. I suggest that the authors further discuss the reasons for choosing these parameters and their correlation with the successful incorporation of ncAAs. Moreover, the description of the screening efficiency is vague. More theoretical justification and experimental evidence should be provided, especially a deeper explanation of the scientific basis for parameter selection, the model training, and the validation process.

2. One limitation of the work is that the ΔG calculation was based on WT aaRS docking. Some ncAAs are clearly unlikely to dock successfully with wild-type aaRS and would require mutations to achieve favorable ΔG values. I am wondering what if the virtual screener fails to predict the incorporation of ncAAs that might otherwise succeed with aaRS mutants? I believe the evaluation metrics need to be discussed.

3. In Figures 3C, 4D, and 4F, the authors only show intensity values. It is unclear how the suppression efficiency compares to wild-type aaRS (%WT). I recommend that the authors provide additional data to address this.

4. In Figure 4F, only two data groups are presented, which makes one-way ANOVA inappropriate. I suggest that the authors correct this.

Author's Response to Peer Review Comments:

For clarity, the original comments for each referee are shown in bold.

Reviewer: 1
Recommendation: Publish in ACS Central Science after minor revisions noted.
Comments:
This manuscript presents a computational model to evaluate the recognition potential of candidate ncAAs by four widely used orthogonal aaRSs. Overall, the work is original, and the model is valuable. It can be published with minor revisions.
We really appreciate the reviewer taking the valuable time to evaluate our manuscript and providing the supportive comments.

1. It is well known that PylRS prefers amino acids with hydrophobic side chains. Therefore, it is not surprising that PylRS does not recognize the negatively charged MalK and GluK, even without the computational model. These examples do not effectively support the authors' claim.

Thank you for pointing this out. We agree with the reviewer and have revised the text to make this clearer (red text on page 3). Therefore, ncAAs designed to mask negative charges are the alternative route to incorporate MalK and GluK. Our model allows for a virtual assessment of the recognition potential of these designed ncAAs for downstream selection by aaRS, thus reducing the need for trial-and-error chemical synthesis.

2. For Figures 3C and 4D, a control-such as wild-type or a reported mutant-should be included for comparison.

As suggested, we have performed the control experiments and replotted the figures (Figure 3C, 4D and Supplementary Figure 3). Thank you!

3. Intuitively, a computational method that aids in the design of aaRSs to recognize new ncAAs would be more valuable than a program that simply advises against proceeding due to potential failures with existing and commonly used protein engineering approaches.

We fully agree with the reviewer's insightful comments and have briefly discussed this topic in the conclusion section.

Regarding the computational design of aaRS for the recognition of new ncAAs, the key issue is the lack of a large amount of reliable data. Existing data on ncAAs and corresponding aaRS mutations (training data) are insufficient to support aaRS design for deep learning. We believe that our approach will greatly facilitate the design, incorporation and application of ncAAs, allowing more and more labs to identify their own ncAAs, and thus facilitating the computational design of aaRS in the future.

4. Nevertheless, it would be beneficial if the authors could outline how they plan to make the model accessible to other researchers.

Thank you for your suggestion. We have uploaded our virtual screening code and stepby-step protocol to Zenodo (https://zenodo.org/records/14043541) so that other researchers can easily use our model in their study.

Additional Questions:
Quality of experimental data, technical rigor: High
Significance to chemistry researchers in this and related fields: High
Broad interest to other researchers: High
Novelty: High

Reviewer: 2
Recommendation: Publish in ACS Central Science after minor revisions noted.
Comments:
Noncanonical amino acids (ncAAs) mutagenesis is an invaluable tool in proteinbased research and therapeutics. One of the current limitations is the structural diversity of sidechains that can be genetically encoded by engineered aminoacyl tRNA synthetase (aaRS). To this end, this manuscript by Fang, et al. developed a virtual screener to determine the recognition potential of ncAAs by analysis and modeling of the side chain structures. Well, most other groups are struggling with computationally designed enzymes to recognize a synthesized ncAAs, this work provide an alternative strategy to virtually screen the recognition potential prior to chemical synthesis. Overall, I find that the manuscript very interesting and would be suitable for publication in ACS Central Science. However, some minor concerns need to be address first.
We really appreciate the reviewer for reviewing our paper and providing insightful and supportive comments.

1. Please define the detailed scientific rationale for parameter selection. Currently, the development of the virtual screening model is primarily based on the permeability, solubility, and binding free energy (ΔG) of ncAAs with aaRS. However, the scientific rationale behind the selection of these parameters is not thoroughly explained. I suggest that the authors further discuss the reasons for choosing these parameters and their correlation with the successful incorporation of ncAAs. Moreover, the description of the screening efficiency is vague. More theoretical justification and experimental evidence should be provided, especially a deeper explanation of the scientific basis for parameter selection, the model training, and the validation process.
Thank you for your insightful and constructive comments. We have revised the manuscript to include a detailed description of the scientific rationale for parameter selection and the model training (red text on page 2). The detailed explanation is as follows and has been included in the revised manuscript:
(1) Scientific rationale for parameter selection
Enzyme kinetics of PylRS and TyrRS have shown that the Km value of ncAA is at the mM range.[1-3] Therefore, selection of active aaRS mutants is only possible if the concentration of the designed ncAA reaches the mM range. The intracellular concentration of a compound depends on its permeability and solubility. Previous data has shown that the intracellular concentration of metabolites is positively correlated with the predicted LogP.[4] For ncAA, the process of genetically encoding 2,3diaminopropionic acid (DAP) further supports our choice of parameters.[5]
Solubility is also critical to maintaining the bioavailability of ncAAs in aqueous environments. Although ncAA is highly permeable, poor solubility can still limit its intracellular concentration, thereby reducing the chance of being incorporated.
The binding affinity (ΔG) between ncAA and aaRS should be in a range that is too weak to be bound by aaRS and too strong to efficiently release the aminoacylation product.
The selected parameters are not independent but synergistically affect the overall success of ncAA incorporation. Therefore, we chose all three parameters to evaluate the key molecular properties required for the ncAA recognition.

(2) Model Development and Training Process
The model was trained using a comprehensive dataset of reported ncAAs including both recognizable and unrecognizable ncAAs from multiple aaRS systems (PylRS, chPheRS, EcTyrRS,

and EcLeuRS). Each ncAA was characterized by its Log P, Log S, and ΔG values, and the dataset was considered as a training set for model building.

3) Validation of the Model
In the model validation, we applied our model to design and incorporate new ncAAs including meMalK, meGluK, and ncAAs with higher negative electrostatic potential. The performance of Prs-GluK in the model further demonstrated the feasibility of our method.

2. One limitation of the work is that the ΔG calculation was based on WT aaRS docking. Some ncAAs are clearly unlikely to dock successfully with wild-type aaRS and would require mutations to achieve favorable ΔG values. I am wondering what if the virtual screener fails to predict the incorporation of ncAAs that might otherwise succeed with aaRS mutants? I believe the evaluation metrics need to be discussed.

We apologize for the poor readability of this part. We have revised the description in Methods to make it clearer. The ΔG calculations are not based on WT aaRS, but on mutants with deep substrate binding pockets. As noted by the reviewer, most ncAAs are unlikely to dock successfully with wild-type aaRS. With the deep substrate binding pocket, all the ncAAs can adopt a suitable pose in the substrate binding pocket and thus give reasonable ΔG values upon docking. These ΔG values can thus reflect the relative binding potential of each ncAA in the deep pocket.

3. In Figures 3C, 4D, and 4F, the authors only show intensity values. It is unclear how the suppression efficiency compares to wild-type aaRS (%WT). I recommend that the authors provide additional data to address this.

As suggested, we have performed the control experiments and replotted the figures (Figure 3C, 4D and Supplementary Figure 3). In the revised paper, the suppression efficiency has been normalized to GFP-WT as 1. Thank you!

4. In Figure 4F, only two data groups are presented, which makes one-way ANOVA inappropriate. I suggest that the authors correct this.

Thank you for pointing this out. The t-test was performed on the data. We have revised the text to make this clear.

Additional Questions:
Quality of experimental data, technical rigor: Top 5%
Significance to chemistry researchers in this and related fields: Top 1%
Broad interest to other researchers: Top 1%
Novelty: Top 1%

References:

1. Guo, L. T.; Wang, Y. S.; Nakamura, A.; Eiler, D.; Kavran, J. M.; Wong, M.; Kiessling, L. L.; Steitz, T. A.; O'Donoghue, P.; Soll, D., Polyspecific pyrrolysyl-tRNA synthetases from directed evolution. *Proc. Natl. Acad. Sci. U. S. A.* **2014,** *111*, 167249.

2. Rauch, B. J.; Porter, J. J.; Mehl, R. A.; Perona, J. J., Improved Incorporation of Noncanonical Amino Acids by an Engineered tRNA(Tyr) Suppressor. *Biochemistry* **2016,** *55*, 618-28.
3. Suzuki, T.; Miller, C.; Guo, L. T.; Ho, J. M. L.; Bryson, D. I.; Wang, Y. S.; Liu, D. R.; Soll, D., Crystal structures reveal an elusive functional domain of pyrrolysyl-tRNA synthetase. *Nat. Chem. Biol.* **2017,** *13*, 1261-1266.
4. Chen, L. L.; Yao, J.; Yang, J. B.; Yang, J., Predicting MDCK cell permeation coefficients of organic molecules using membrane-interaction QSAR analysis. *Acta Pharmacol Sin* **2005,** *26*, 1322-33.
5. Huguenin-Dezot, N.; Alonzo, D. A.; Heberlig, G. W.; Mahesh, M.; Nguyen, D. P.; Dornan, M. H.; Boddy, C. N.; Schmeing, T. M.; Chin, J. W., Trapping biosynthetic acyl-enzyme intermediates with encoded 2,3-diaminopropionic acid. *Nature* **2019,** *565*, 112-117.
